# The Best-Practice Organism for Single-Species Studies of Antimicrobial Efficacy against Biofilms Is *Pseudomonas aeruginosa*

**DOI:** 10.3390/membranes10090211

**Published:** 2020-08-30

**Authors:** Anna M. Curtin, Matthew C. Thibodeau, Heather L. Buckley

**Affiliations:** Green Safe Water Lab, Civil Engineering Department, University of Victoria, Victoria, BC V8P 5C2, Canada; annacurtin@uvic.ca (A.M.C.); matthew.thibodeau@outlook.com (M.C.T.)

**Keywords:** reverse osmosis, biofouling, single-species, prevention, semi-systematic review

## Abstract

As potable water scarcity increases across the globe; it is imperative to identify energy and cost-effective processes for producing drinking-water from non-traditional sources. One established method is desalination of brackish and seawater via reverse osmosis (RO). However, the buildup of microorganisms at the water-membrane interface, known as biofouling, clogs RO membranes over time, increasing energy requirements and cost. To investigate biofouling mitigation methods, studies tend to focus on single-species biofilms; choice of organism is crucial to producing useful results. To determine a best-practice organism for studying antimicrobial treatment of biofilms, with specific interest in biofouling of RO membranes, we answered the following two questions, each via its own semi-systematic review: 1. Which organisms are commonly used to test antimicrobial efficacy against biofilms on RO membranes? 2. Which organisms are commonly identified via genetic analysis in biofilms on RO membranes? We then critically review the results of two semi-systematic reviews to identify pioneer organisms from the listed species. We focus on pioneer organisms because they initiate biofilm formation, therefore, inhibiting these organisms specifically may limit biofilm formation in the first place. Based on the analysis of the results, we recommend utilizing *Pseudomonas aeruginosa* for future single-species studies focused on biofilm treatment including, but not limited to, biofouling of RO membranes.

## 1. Introduction

Across the globe, there are increasingly inadequate amounts of clean water to meet human and environmental needs. For example, as of 2017, 20% of the global population lacked clean drinking water [1]. This perilous situation is largely due to increasing global populations and increasing demands for water, as well as changing climate patterns. Fortunately, we have not yet effectively utilized non-traditional water sources, such as natural and human-caused brackish water sources (i.e., saltwater intrusion in overused groundwater aquifers) and seawater, which if treated could provide water for many. One way to treat this water is via reverse osmosis (RO). RO systems use an external force to push water across a semipermeable membrane from the feed side containing solutes to the permeate side containing potable water [2].

RO water treatment is a membrane-based technology that can enable the use of typically non-potable water sources, ranging from brackish water (1000–10,000 ppm) to seawater (10,000–35,000 ppm) [3]. Desalination via RO, however, is limited significantly by fouling of the RO membranes [4,5,6,7,8]. The most significant form of fouling is biofouling, which is involved in more than 45% of RO membrane fouling [4,8,9,10] Biofouling occurs when a biofilm forms on membrane material at the water-membrane interface. A biofilm is a complex of microorganisms, including bacteria, fungi, and algae, and extracellular polymeric substances (EPS) [11,12,13]. The process of biofilm formation proceeds as follows: (1) conditioning of the membrane by EPS secreted by microorganisms or from the bulk water, (2) attachment of pioneer microorganisms, which are the organisms that condition the membrane and are the first to attach to it; (3) diversification, growth, and metabolism of attached microorganisms, and (4) development into a mature biofilm [12,13,14]. Mature biofilms consist of up to 90% EPS by mass [8,13].

**Figure 1:**
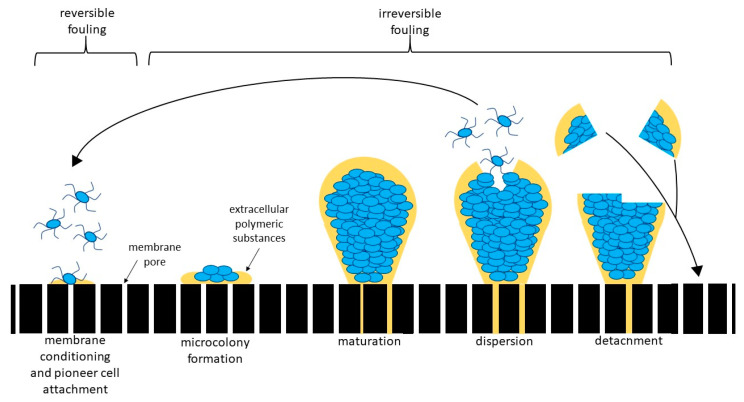
Biofouling occurs via a four-step process: (1) conditioning of the membrane by pioneer organisms or material in the bulk water, (2) attachment of pioneer organisms to the conditioned surface, (3) formation of microcolonies, and (4) formation of a mature biofilm consisting of a community of organisms in a matrix of extracellular polymeric substances (EPS) [26]. Up to 90% of the biofilm consists of the EPS matrix, most produced by the bacteria with in it, with the remaining 10% consisting of the bacterial organisms [9]. Before microcolony formation, biofouling is considered reversible fouling because it can be removed by shear force. However, after microcolony formation, biofilms are too strongly attached to the membrane to detach with increased shear force, therefore, requiring other treatment methods (i.e., chemical treatment).

The steps of biofouling can be broken up into two categories: reversible and irreversible fouling. Reversible biofouling consists of loosely deposited or bound foulants on the membrane, which can be removed by backwashing the system or increased shear force [15]. Reversible fouling occurs before microcolony formation (Figure 1). Reversible fouling is still detrimental because it can result in temporarily stopping the production of potable permeate in order to backwash the system. The other type is irreversible biofouling, which includes biofilms from microcolony formation to mature biofilms in a matrix of EPS [16] (Figure 1). It cannot be removed by backwashing or increasing flux; this means the membrane needs to be removed and chemically treated or completely replaced. Often, chemical treatments, such as chlorine, damage the membrane, decreasing the membrane lifespan [17].

The low permeability of the biofilm causes membrane flux to decrease, which then requires increased energy input to maintain flux [9]. The accepted fouling model suggests that fouling effects flux in two phases [16,17]. During the first phase, flux decreases rapidly due to the compaction of the membrane and the development of irreversible fouling. During the second phase, the rate of decrease in flux is smaller due to fouling, reaching a state of homeostasis between biofilm formation and sloughing off of the mature biofilm. The biofilm can also lead to the biodegradation or corrosion of the membrane due to acidic byproducts [9]. In addition, the torturous path of the heterogeneous structure of the biofilm inhibits back diffusion, which can lead to an increased degree of concentration polarization, leading to the increased passage of salt across the membrane [18]. This phenomenon is known as biofilm enhanced osmotic pressure (BEOP). Finally, anti-fouling treatment methods can wear down membranes, which shortens their lifespan [12]. Ultimately, biofouling decreases the efficiency of RO membranes, consequently increasing operational and maintenance costs for water treatment plants [18,19,20,21,22,23]. These drawbacks cause RO to be disadvantageous for promoting cost-effective, sustainable communities and cities.

Common treatment methods are often reactive, meaning they attempt to treat biofouling after mature biofilms have formed [9]. Treating mature biofilms is difficult for many reasons. Firstly, physical cleaning methods are ineffectual or near impossible on spiral-wound membranes [24], which are the most common type of membrane used in RO systems [9]. The structure of mature biofilms also protects microorganisms from chemical and physical treatment methods. Additionally, microorganisms in biofilms experience enhanced gene transfer, which allows microorganisms to share beneficial traits, such as antimicrobial resistance, which increases the resistance of mature biofilms to treatment [14]. The protective mechanisms facilitated by the microenvironment of a mature biofilm, especially increased antibiotic resistance, make biofouling one of the most difficult forms of fouling to treat [14]. Biofouling is also considered the “Achilles heel” of RO because even if 99.9% of the microorganisms are removed from the membrane, the remaining microorganisms can re-form a biofilm [9]. Moreover, treating biofilms is a complex problem because the components of biofilms vary depending on the type of microorganisms in the water and on the environmental and operational conditions [25].

Various prevention and treatment methods are being studied to attempt to mitigate biofouling, including chemical treatments (i.e., linoleic acid (plant fatty acid) [27]; nitric oxide [28]; urea [29]), membrane modifications (i.e., silver nanoparticles [30,31,32,33]; triclosan [34]; carbon nanotubes [35]; Arabic gum [36,37]; hydrophilic membranes [38]; capsaicin [39]), and biological treatments (i.e., bacteriophages [40]; quorum-quenching [41,42]). Before these methods can be employed, the treatment methods must be rigorously tested to determine anti-biofouling efficacy, impacts on the membranes, and safety for humans and the environment. Selecting appropriate organisms to test efficacy is integral, especially since studies are often performed on single-species biofilms, due to the complexities of multi-species biofilms [43]. Emphasis should be put on organisms that are integral to biofilm formation, namely, pioneer organisms. If treatment methods focus on preventing pioneer organisms from surviving or producing EPS, a biofilm could potentially be avoided.

One way to identify common biofilm organisms is via genetic analysis [43]. If samples are collected early on in biofilm formation, pioneer organisms can be identified. The genetic analysis includes genotypic methods for identifying bacteria, which are more accurate than other common phenotypic methods of identification, i.e., morphological characteristics [43]. The most common method of genetic analysis of bacteria is 16S ribosomal RNA (rRNA) gene sequencing. First, researchers extract DNA from samples. Extraction methods range from total nucleic acids [43,44,45] via kits, such as the Soil Master™ DNA extraction kit [46], to the isolation of bacteria (i.e., via agar spread plates) and purification based on macro and micro-morphology [47,48]. The former method samples all organisms, and the latter samples only organisms that are culturable; therefore, it leaves out viable but not culturable organisms. Next, researchers use primers homologous to portions of the 16S rRNA gene to amplify the DNA via polymerase chain reactions (PCR) [47,48,49,50,51,52]. The amplified DNA is then sequenced, and the sequences are processed, after which organisms can be identified using databases, such as EzTaxon [21] or NCBI Blast [53]. This identification method can lead to operational taxonomic unit (OTU) classifications when strains have less than 97–98% similarity; however, above 97% similarity, organisms must be differentiated via alternative approaches, such as DNA-DNA hybridization [51,52,53].

Researchers sequence fragments of the 16S rRNA gene because the gene codes for the RNA component of the 30S subunit of the bacterial ribosome, which is present in all bacterial species [54]. Additionally, the gene has multiple highly conserved regions with nine variable regions dispersed throughout. Researchers can design primers homologous to the conserved regions, which will lead to the amplification of portions of the gene that include the variable regions that are used to distinguish species [55,56]. Another benefit of 16S rRNA gene sequencing is the abundance of 16S sequence data available for bacterial organisms. As stated earlier, the major limitation of 16S rRNA gene sequencing is the inability to resolve species classification of strains with too high similarities [54]. For example, Janda and Abbott [57] found that 16S rRNA was able to determine the genus classification of 90% of isolates; however, it was only able to resolve species classification of 65–83% of strains.

This study attempted to answer the question of which organism should be the focus of anti-biofouling studies with an emphasis on biofouling prevention, rather than a mature biofilm removal perspective. We answered this question by performing two semi-systematic reviews to investigate the following related questions:

1. Which organisms are commonly used to test antimicrobial efficacy against biofilms on RO membranes?
2. Which organisms are commonly identified via genetic analysis in biofilms on RO membranes?
3. Based on the results of questions 1 and 2, we additionally answered the following question: Which of the identified organisms are pioneer organisms?

The first question will inform about organisms that are accepted model organisms for biofilm treatment. The answers to question 1 will allow researchers to choose organisms for their studies in such a way that they can compare their results to relevant existing literature. Utilizing an organism identified via question 2 will ensure that the organisms are relevant to biofilms on RO membranes. Finally, utilizing an organism identified in question 3 will be useful for identifying proactive treatments for biofouling. Ultimately, these analyses allow us to recommend a best-practice organism for studying the treatment of biofilms with an emphasis on biofouling of RO membranes, especially biofilm prevention.

## 2. Semi-Systematic Review Methods

The semi-systematic reviews report pertinent information according to the Preferred Reporting Items for Systematic Reviews and Meta-analysis (PRISMA) and are guided by the reporting style in Cassivi et al. [58]. All authors agreed to the semi-systematic review protocol. Study selection was completed by (AC and MT). The Web of Science© database was used to identify peer-reviewed literature that satisfies the semi-systematic review criteria discussed below. We used the term semi-systematic review because only one database was utilized to collect articles [59].

### 2.1. Semi-Systematic Review: Which Organisms Are Used for Anti-Biofouling Studies?

#### 2.1.1. Eligibility Criteria

Studies that utilized bacterial organisms to test antimicrobial efficacy against biofilms on RO water treatment membranes.

#### 2.1.2. Search Strategy

The following Boolean search terms were used for the semi-systematic review: ‘(reverse osmosis OR RO) AND (bio-fouling OR biofouling OR biofilm) AND (anti-microbial OR antimicrobial OR anti-bacterial OR antibacterial OR anti-fouling OR antifouling OR anti-foulant OR antifoulant OR bacteriostat * OR bactericid *)’ AND water’.

#### 2.1.3. Study Selection

**Figure 2:**
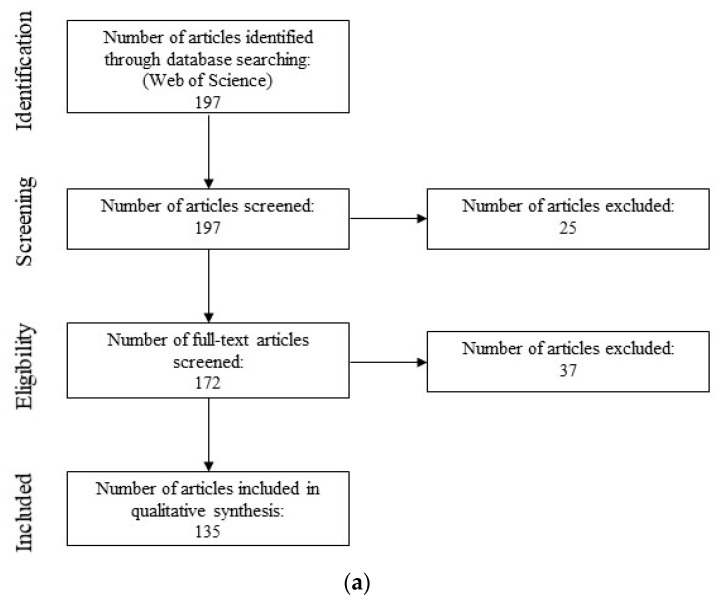

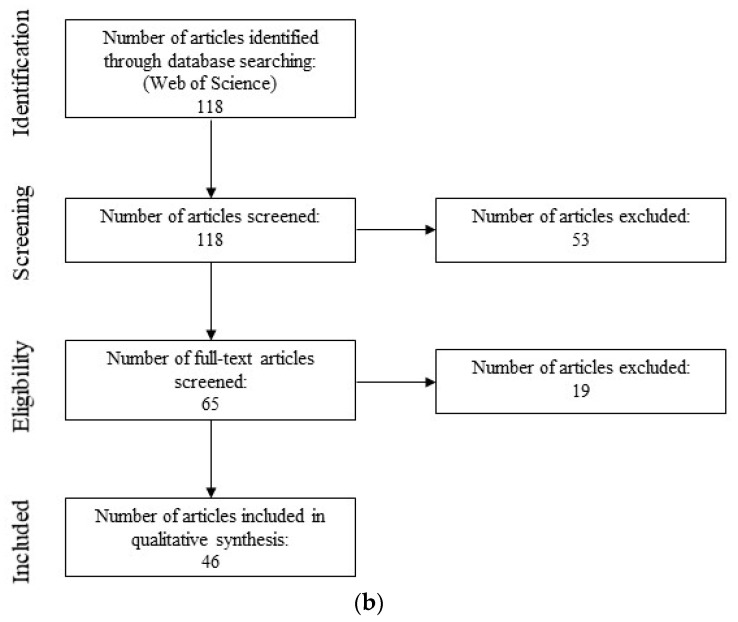
Flow chart of the selection process for articles via the semi-systematic review for Section 2.1 (**a**) and Section 2.2 (**b**), similar to Moher et al. [60].

Studies were selected following the PRISMA flow chart reported in Moher et al. [60]. The final Boolean search terms resulted in 197 articles, which were exported to Mendeley (Figure 2a). The abstracts of these articles were analyzed to determine which were related to the purpose of the semi-systematic review, which resulted in the removal of 25 articles. For the remaining 172 articles, the entire article was analyzed to determine whether it contained primary studies related to the purpose of the semi-systematic review. During this step, 37 articles were removed, including 13 review articles, 12 articles that used real water samples that were not analyzed to identify bacterial organisms, and 12 articles that did not include necessary content (i.e., not about RO).

The final analysis, therefore, included 135 of the articles identified via the semi-systematic review (Supplementary Information Table S1). The articles included studies that tested antimicrobial chemicals and membrane modifications in experiments ranging from biofilms in 96-well plates to bench-scale RO systems. Some articles included antimicrobial tests against suspension cultures (planktonic phase cells) prior to biofilm tests. We included all organisms used in those studies, even if some organisms were only used for the suspension culture aspect of the paper. We chose to include these because even though the organisms were not grown as biofilms, the data were still used to inform subsequent tests against biofilms.

#### 2.1.4. Data Collection, Extraction, and Analysis

The 135 articles were analyzed to extract pertinent information, including general information (title, publication date, journal name), antimicrobial type (chemical, heavy metal, membrane modification, other), organism(s) tested, the organism phase (suspension culture, agar plate, or biofilm), biofilm detection method (i.e., LIVE/DEAD BacLight Stain, SEM, colony counting, measuring membrane flux, etc.), and whether or not the paper mentioned that the test bacteria was chosen because it is a model organism.

### 2.2. Semi-Systematic Review: Which Organisms Are Found in Biofilms on RO Membranes?

#### 2.2.1. Eligibility Criteria

Studies that identified organisms in biofilms on RO membranes via 16S rRNA gene sequencing.

#### 2.2.2. Search Strategy

The following Boolean search terms were used for the semi-systematic review: ‘(common OR pioneer OR divers * OR ((early AND coloniz *) OR (first AND coloniz *)) OR microb * analysis OR DNA analysis OR genetic analysis) AND (bacteria * OR organism * OR species OR microorganism OR microb *) AND (biofilm OR bio-fouling OR biofouling) AND (RO OR reverse osmosis) AND water’.

#### 2.2.3. Study Selection

Studies were selected following the PRISMA flow chart reported in Moher et al. [60]. The final Boolean search terms resulted in 118 articles, which were exported to Mendeley (Figure 2b). The abstracts of these articles were analyzed to determine which were related to the purpose of the semi-systematic review, which resulted in the removal of 53 articles. For each remaining study, the entire article was analyzed to determine whether it related to the purpose of the semi-systematic review. During this step, 19 articles were removed, including 4 review articles, 5 articles that did not directly relate to RO or water treatment, and 5 articles that did not include a genetic analysis component.

The final analysis, therefore, included 46 of the articles identified via the semi-systematic review (Supplementary Information Table S2). The articles included in the study performed 16S rRNA gene sequencing on RO biofouling samples retrieved from full-scale desalination plants, pilot-scale systems, and bench-scale systems. The feedwater sources included seawater, wastewater treatment plant influent and effluent, industry wastewater, membrane bioreactor effluent, tap water, drinking water, and well water. We recorded the most specific taxonomical classification provided for each organism ranging from phyla to species level.

#### 2.2.4. Data Collection, Extraction, and Analysis

The 46 articles were analyzed to extract pertinent information, including general information (title, publication date, journal name), feed water source(s) (i.e., seawater, wastewater treatment plant, etc.), level of taxonomical identification, and bacteria identified. We also analyzed the articles for mentions of pioneer organisms.

### 2.3. Phylogenetic Tree-Like Structure

A phylogenetic tree-like structure was constructed using the NCBI Taxonomy Database and Common Tree, a tool created to show a “hierarchical view of the relationships among the taxa and their lineages” [61,62,63]. The tree consisted of identifications from semi-systematic review 1. Due to the quantity of identified organisms, the tree was limited to class level identifications. The tree was viewed and manipulated using ggtree [64,65].

## 3. Results

### 3.1. Semi-Systematic Review: Which Organisms Are Used for Anti-Biofouling Studies?

**Figure 3:**
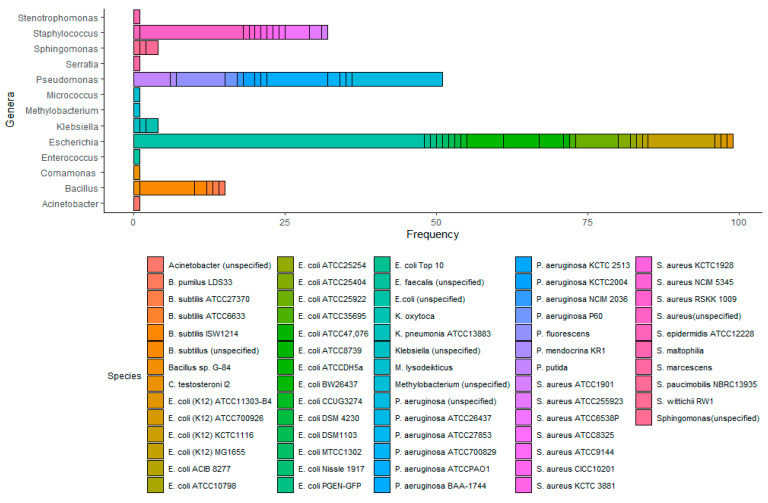
Frequency, in terms of the number of articles where the organism occurs, of use of microorganisms to study antimicrobial efficacy against biofilms on RO membranes cited in the literature. Data were acquired from an analysis of 135 articles related to biofouling in water treatment systems.

One-hundred-and-thirty-five articles contributed to a consensus from the literature of common microorganisms used to test antimicrobial efficacy against biofilms on RO membranes. Upon analyzing these articles, thirteen genera were identified in at least one article, including *Acinetobacter* (1 occurrence), *Bacillus* (15), *Comamonas* (1), *Escherichia* (99), *Enterococcus* (1), *Klebsiella* (4), *Micrococcus* (1), *Methylobacterium* (1), *Pseudomonas* (51), *Serratia* (1), *Staphylococcus* (32), *Stenotrophomonas* (1), and *Sphingomonas* (4), resulting in a total of 212 organisms used in the 135 studies (Figure 3). Of the analyzed literature, approximately 93% of the identified organisms came from four different genera, including *Escherichia*, which was used in 73% of the articles, *Pseudomonas*, which was used in 38% of the articles, *Staphylococcus*, which was used in 24% of the articles, and *Bacillus*, which was used in 11% of the articles. The remainder of the genera (5% of all of the identified organisms) occurred in less than 3% of the articles each.

The most commonly used strain was *Escherichia coli* (*E. coli*) K12 MG1655, which was used in 11 articles, followed by *Pseudomonas aeruginosa* (*P. aeruginosa*) ATCC PAO1, which was used in 10 articles. We also found that 73% of the articles included in the final analysis stated that the organism(s) they used was model or typical organisms or simulated biofouling. Five genera were not described in any of the articles as model organisms, including *Acinetobacter*, *Methylobacterium*, *Stenotrophomonas*, *Serratia*, and *Sphingomonas*.

### 3.2. Semi-Systematic Review: Which Organisms Are Found in Biofilms on RO Membranes?

**Figure 4:**
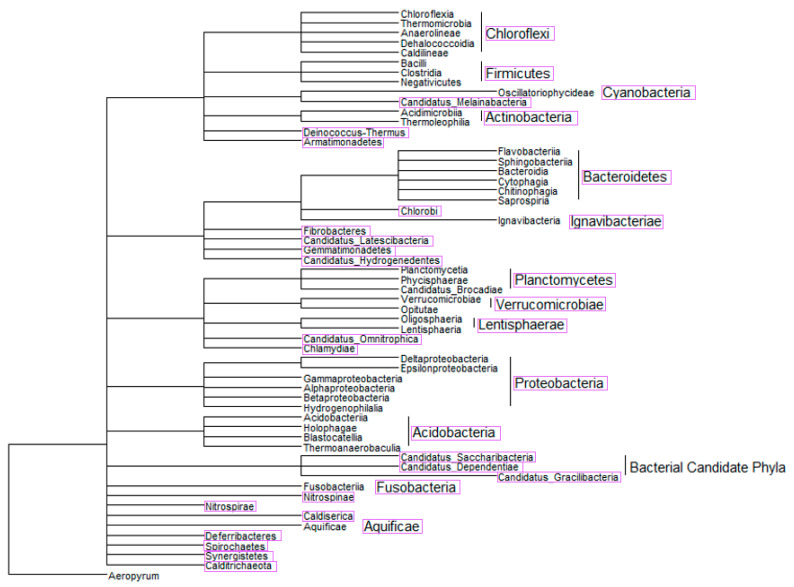
A phylogenetic tree-like structure was constructed of the organisms identified in semi-systematic review 1 down to class level (outlined in pink) to show the diversity of organisms that are found in biofilms on RO membranes. The tree-like structure shows hierarchal clusters between organisms based on data in the NCBI Taxonomy Database and Common Tree [61,62,63,64,65].

**Figure 5:**
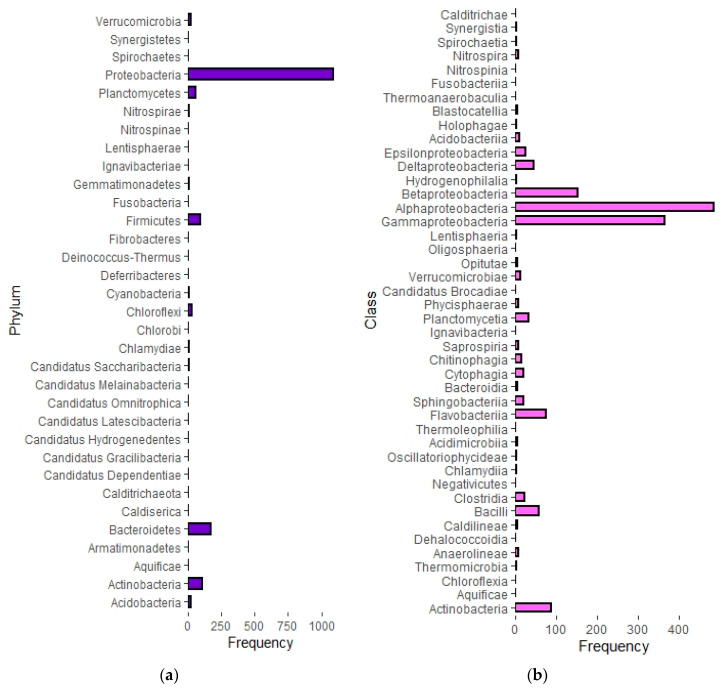
Phylum-level frequency of organisms identified through semi-systematic review 1 (**a**) Class-level frequency of organisms identified through semi-systematic-review. (**b**) The frequency is based on the number of times an organism from a specific phylum or class was identified in the analyzed literature.

Figure 4 shows the constructed phylogenetic tree, displaying the class and phyla diversity of the organisms that were identified in the analyzed literature. Forty-four classes were identified that corresponded to thirteen phyla, and an additional twenty phyla were identified but not resolved down to class level. Figure 5a shows the frequency with which organisms were identified in the thirty-three phyla, and Figure 5b shows the frequency organisms were identified in the forty-four classes.

Of the phyla, organisms in *Proteobacteria* (1090 identifications) accounted for 66% of all the identified organisms, corresponding to six times more organisms than the next highest phylum, *Bacteroidetes* (171 identifications). Following *Bacteroidetes*, were *Actinobacteria* (110 identifications), *Firmicutes* (90 identifications), and *Planctomycetes* (55 identifications). The remaining phyla combined accounted for only 7% of the identified organisms. At the class level, 32% of the identified organisms were part of the class *Alphaproteobacteria*, 24% in *Gammaproteobacteria*, 10% in *Betaproteobacteria*, 6% in *Actinobacteria*, 5% in *Flavobacteriia*, and 4% in *Bacilli*. The remaining 38 classes of organisms made up less than 20% of the total occurrences.

## 4. Discussion

### 4.1. Which Organisms Are Used for Anti-Biofouling Studies?

To choose successful antifoulants, studies against single-species biofilms should be performed against model biofilm-forming organisms. If tests are performed against model organisms, the results will be more representative of actual efficacy.

In this analysis, we found that many of the organisms used in the studies were chosen because they are considered model organisms. For example, *P. fluorescens* was used in [66] because it was considered convenient and a relevant model organism for biofilm formation. Similarly, [67] describes that *Pseudomonas* spp. are useful model organisms for studying biofouling based on the secretion of EPS. Additionally, Zhu et al. [68] stated that *E. coli* and *P. aeruginosa* were used in their study because they are commonly used as model bacteria in antibacterial studies. Only five of the genera, including *Acinetobacter*, *Methylobacterium*, *Stenotrophomonas*, *Serratia*, and *Sphingomonas*, have not been specifically described as model organisms in any of the analyzed literature; however, this does not mean that they are not model organisms. For example, *Sphingomonas* spp. are recommended as a model organism for biofouling, especially for studying initial attachment and growth of biofilms [69].

This analysis identified genera that contain model organisms that are commonly used to test antimicrobial efficacy against biofilms on RO membranes, which would provide relevant organisms for antimicrobial efficacy studies. However, there are some limitations to its application. Firstly, our analysis included organisms that were only tested in the suspension culture phase in a biofilm study. We included these organisms because we wanted to include all organisms that were used to inform about biofilms, not only the ones specifically grown as biofilms. The most common organism in our study, where this was the case, was *E. coli.* For example, Flemming and Wingender [70] noted that they did not use their *E. coli* strain for some of the biofilm tests they performed because it formed a mature biofilm very slowly and did not allow for clear comparisons between adhered cells and non-adhered cells. Therefore, although the researchers were not able to gather results for *E. coli* biofilms, they intended to use *E. coli* as a model biofilm organism. However, due to this phenomenon, we investigated concerns with *E. coli* biofilm formation. In Narisawa et al. [71], the researchers found that there were 10-fold less *E. coli* W3110 cells incorporated in the biofilm compared to *E. coli* IAM1264, corresponding to 1.62 × 10^6^ and 1.74 × 10^7^ cells/well, respectively, highlighting the importance of strain selection. According to Spoering and Lewis [72], *P. aeruginosa* PAO1 biofilms contained about 10^8^ cells/well in the biofilms, suggesting *P. aeruginosa* could lead to denser biofilms than the *E. coli* strains used in Narisawa et al. [71], which may explain why *E. coli* can lead to unsatisfactory biofilms (i.e., [70]). Cell density is especially important because biofilm detection methods, such as Crystal Violet stain, require sufficient biomass for accurate measurements [73].

Another possible limitation of this analysis is the inherent focus on opportunistic pathogens in biofilm research. For example, Davies [74] stated that many studies related to biofouling in water treatment-related systems focus on opportunistic pathogens, including *Pseudomonas*, *Staphylococcus*, and *Escherichia*. An opportunistic pathogen is an organism that normally has a commensal relationship with a host but can infect hosts under certain circumstances, such as individuals that have compromised immune systems. The organisms, therefore, are a public health concern, which warrants increased attention in the literature; however, these organisms may not be the most relevant from the perspective of biofouling on RO membranes. This could account for the high incidence of use of *E. coli* in 68% of the articles and *P. aeruginosa* in 35% of the articles. However, this is likely not a concern because one quality of opportunistic pathogens is the ability to form biofilms [74,75,76,77]. Additionally, *Pseudomonas* and *Staphylococcus* are specifically identified as opportunistic pathogens that are also biofilm formers in López et al. [78].

### 4.2. Which Organisms Are Found in Biofilms on RO Membranes?

This semi-systematic review for question 2 determined thirty-three phyla of organisms corresponding to forty-four classes of organisms that were identified in various studies via 16S rRNA analysis in biofouling samples from RO systems. Similar to results from question 1, the most common organisms identified were in the phylum *Proteobacteria* [21,44,79]. The top three most frequent classes of organisms were in *Proteobacteria*, including, *Alphaproteobacteria*, followed by *Gammaproteobacteria* and *Betaproteobacteria*. These results were supported by results found in [21,44,77,79,80]. For example, in Hörsch et al. [81], researchers found *Alphaproteobacteria* to be the most abundant class of organisms on RO membranes, followed by *Gammaproteobacteria*. Ivnitsky et al. [80] found *Gammaproteobacteria* to be the most abundant organisms; however, the author stated that after more than thirty days of activity, the biofilm became dominated by *Alphaproteobacteria* and *Betaproteobacteria*, which could explain why our study determined more *Alphaproteobacteria* than *Gammaproteobacteria* when analyzing the data from all of the studies that include data collection at multiple time points [80].

The next most common phylum was *Bacteroidetes*. Ferrera et al. [82] stated that *Bacteroidetes* was found in biofilms in samples taken at one and three months; thus, organisms in *Bacteroides* are considered regular members of biofilms on RO membranes, but not necessarily pioneer organisms [28,83,84,85]. *Bacteroidetes* contain the second most common class of organisms, *Flavobacteriia*, and the eighth and ninth most common classes, including *Cytophagia* and *Sphingobacteriia*, respectively. All three of these classes are commonly found in mature biofilms on RO membranes [69,86,87].

The phylum *Actinobacteria* is the next most common, which includes the most common class, *Actinobacteria*. Studies have found that although early on the biofilms tend to include *Actinobacteria*, their abundance decreases over time [88,89]. The phylum *Firmicutes* closely follows *Actinobacteria* in frequency. It contains the third most common class, *Bacilli*, and the seventh most common class, *Clostridia*. One study found that the amount of *Firmicutes* increased over time as the mature biofilm formed [89]. *Planctomycetes* is the last phylum of bacteria with a relevantly high frequency. It contains the fifth most common class, *Planctomycetia*. One study found that organisms in *Planctomycetes* were of consistently high abundance throughout biofilm formation [85].

A major reason some of the previously mentioned phyla and classes are commonly found in biofilms on RO membranes is due to the production of EPS. EPS is integral for conditioning of the membrane for initial attachment and for the development of mature biofilms. According to Shang et al. [79], organisms in *Proteobacteria* produced more EPS than other bacterial phyla. For example, Ivnitsky et al. [81] identified that *Gammaproteobacteria* had superior attachment ability compared to other organisms due to the production of EPS, making it a common pioneer organism. Since EPS is so important for biofilms, it likely explains why organisms in *Proteobacteria* were most commonly identified in this study. More specifically, organisms in *Alphaproteobacteria*, *Betaproteobacteria*, *Gammaproteobacteria*, *Bacteroidetes*, and *Actinobacteria* produce amyloid adhesins, which constitute a large fraction of EPS in microcolonies [90]. Amyloids are insoluble and highly tolerant of denaturants, making removal of this EPS difficult [91]. Albertsen et al. [92] analyzed the genes of *Bacteroidetes* and characterized a gene for alginate production, which is another type of EPS. *Firmicutes* produce hydrophobic EPS, which clumps cells and contributes to biofilm formation [91]. Uniquely, the major reason organisms in *Planctomycetes* are believed to be effective biofilm formers is because they are budding bacteria, some of which are filamentous, which leads to aggregation of cells [85]. Organisms in this phylum tend to be found at the base of biofilms, suggesting they participate in early biofilm formation [85].

### 4.3. Which of the Identified Organisms Are Pioneer Organisms?

Of the thirteen genera identified for question 1, eight of the genera, including *Acinetobacter*, *Bacillus*, *Escherichia*, *Methylobacterium*, *Pseudomonas*, *Staphylococcus*, *Stenotrophomonas*, and *Sphingomonas*, all contain species that are considered to be pioneer organisms of biofilms [14,69,89,93,94,95,96]. The first step of biofilm formation on a membrane is conditioning by EPS, which is excreted directly onto the membrane by pioneer organisms or sourced from the bulk water [17]. EPS helps facilitate the attachment of organisms to the membrane in combination with flagella, type I pili, and outer membrane proteins [97]. For example, *P. aeruginosa* produces Pel and Psl, which are both important EPS for attachment to abiotic and biotic surfaces and for initial biofilm formation [70]. If treatment methods are focused on preventing the excretion of EPS and attachment of pioneer organisms, mature biofilms may not be able to form, negating the need for the complex treatment methods that are required for mature biofilms. It should be noted that an extensive EPS matrix, containing pores for water and nutrient flow, is an integral component of a mature biofilm; therefore, EPS is also important in mature biofilms. For example, *P. aeruginosa* produces alginate, which is a type of EPS that is not integral for initial biofilm formation but is important for producing the three-dimensional structure of a biofilm. Without alginate, *P. aeruginosa* biofilms are flat and thin [70].

For question 2, we found that the most common organisms in biofilms on RO membranes were from the phylum *Proteobacteria*. Shang et al. [79] concluded that the phyla *Proteobacteria* contained the main pioneer organism of marine biofilms on RO membranes, which was also supported by results from Ma et al. [98] and Hu et al. [99]. As stated earlier, Hörsch et al. [81] found *Gammaproteobacteria* to be the first colonizers of the membrane. Because *Gammaproteobacteria* are recognized as major pioneer organisms on RO membranes, testing treatment methods on these organisms could provide representative models for biofouling prevention efficacy.

### 4.4. Comparison

Comparing the organisms identified in Section 3.1 to the organisms identified in Section 3.2 suggests that laboratory studies are being performed on organisms that are commonly found on RO membranes, ranging from pioneer organisms forming the biofilm to organisms involved in mature biofilms. The following classes contained pioneer organisms that were commonly used to study the anti-biofouling efficacy of antimicrobials: *Acinetobacter*, *Bacilli*, *Gammaproteobacteria*, and *Alphaproteobacteria*. The previous review found that the most commonly tested organisms were in the class *Gammaproteobacteria* in the genus *Escherichia*. *Escherichia* was likely tested most often because it is a model bacterial organism and because it is a common fecal contamination indicator used in water treatment [100,101]. *Pseudomonas* was identified as the next most commonly used organism in the previous semi-systematic review. It is also in the class *Gammaproteobacteria*. In the case of *Pseudomonas*, we suggest that the use of *Pseudomonas* spp. in anti-biofouling efficacy studies relates to *Pseudomonas* spp. being model biofilm formers that produce EPS, which is an important characteristic of pioneer organisms. *Pseudomonas* spp. also have clinical health relevance due to their pathogenicity, which may contribute to its use in anti-biofouling studies (i.e., [102,103,104]).

We recommend utilizing the *Pseudomonas* spp. instead of *Escherichia* for anti-biofouling studies. One reason is due to concerns with the density of *Escherichia* biofilms, which will affect detectability. Additionally, some *Pseudomonas* species are pioneer organisms, which are useful from a biofilm prevention perspective. Finally, *P. aeruginosa* is an opportunistic pathogen, and therefore results from anti-biofouling studies in regard to RO may have applications in other fields.

## 5. Conclusions

Ultimately, antifouling efficacy tests should be performed on model biofilm-forming pioneer organisms that are commonly found in biofilms on RO membranes. A focus on pioneer organisms could cause results to be more relevant from a prevention perspective. The organisms that most closely fit that criteria are in the class *Gammaproteobacteria*. A common genus in this class that is already used for tests against biofilms is *Pseudomonas*. This genus contains organisms that are considered model biofilm formers as well as pioneer organisms. We recommend utilizing *P. aeruginosa*, which is commonly used for biofilm studies and identified via genetic analysis in biofilms on RO membranes. It should be acknowledged, however, that biofilms are very complex; therefore, single-species studies are only so useful, no matter which organisms are tested. It is integral that tests move beyond focusing on pioneer organisms and focus on the complex communities that make up biofilms to obtain the most realistic results for an anti-biofouling treatment method.

## Supplementary Materials

The following are available online at https://www.mdpi.com/2077-0375/10/9/211/s1, Table S1. Semi-systematic review raw data answering: Which organisms are used for anti-biofouling studies? Table S2. Semi-systematic review raw data answering: Which organisms are found in biofilms on RO membranes?
