# Supplementary material for: The Best-Practice Organism for Single-Species Studies of Antimicrobial Efficacy against Biofilms Is Pseudomonas aeruginosa
Source: Membranes (Basel). 2020 Aug 30;10(9):211. doi: 10.3390/membranes10090211 (PMC7559251; doi:10.3390/membranes10090211)
Supplement: Supplementary file 1 [file membranes-10-00211-s001.pdf]

Table S1. Semi-systematic review raw data answering: Which organisms are used for anti-biofouling studies?

Antifoulant Method	Organism(s)	Model Bacteria (Y if mentioned)	Type of Biofilm Detection Method	Source
composite membranes	E. coli ATCC25922<br><br>S. aureus ATCC255923	Y	LIVE/DEAD baclight stain	[1]
composite membranes	E. coli ATCC25922<br>S. aureus RSKK 1009	Y	colony counting	[2]
graphene oxide	Saccharomycetes		colony counting	[3]
methyl p-hydroxybenzoate<br>potassium sorbate	L. monocytogenes<br>P. putida<br>Y. enterocolitica<br>A. hydrophila			[4]
composite membranes	E. coli (unspecified/unique sample type)<br>S. aureus (unspecified/unique sample type)<br>K. pneumonia ATCC13883<br>P. aeruginosa BAA-1744	Y	FESEM	[5]
composite membranes	E. coli (unspecified/unique sample type)<br>S. aureus (unspecified/unique sample type)	Y	SEM	[6]
graphene oxide	E. coli ATCC25922<br>S. aureus ATCC9144<br>P. aeruginosa ATCCPAO1	Y	colony counting	[7]
composite membranes	E. coli (unspecified/unique sample type)	Y	measuring flux	[8]
graphene oxide	E. coli (unspecified/unique sample type)<br>S. aureus (unspecified/unique sample type)	Y	colony counting<br>SEM<br>LIVE/DEAD baclight stain	[9]
modified membrane	P. aeruginosa P60<br>Bacillus sp. G-84	Y	DAPI<br>LIVE/DEAD baclight stain	[10]
bacteriophages	E. coli (K12) ATCC11303-B4	Y	measuring flux	[11]
quorum quenching	P. aeruginosa KCTC 2513		LIVE/DEAD baclight stain	[12]
modified membrane	E. coli (unspecified/unique sample type)<br>S. aureus (unspecified/unique sample type)		colony counting<br>colony counting<br>measuring flux	[13]
modified membrane	E. coli BW26437	Y	measuring flux	[14]

graphene oxide	Klebsiella (unspecified/unique sample type)<br>P. aeruginosa (unspecified/unique sample type)		colony counting	[15]
graphene oxide	P. aeruginosa (unspecified/unique sample type)		measuring flux	[16]
composite membranes<br>modified membrane	E. coli (K12) MG1655		DAPI<br>PI	[17]
composite membranes	E. coli (unspecified/unique sample type)		counting fluorescent cells	[18]
modified membrane	E. coli (K12) MG1655	Y	PI<br>DAPI<br>colony counting	[19]
modified membrane	B. subtilis ISW1214<br>M. lysODeikticus	Y	colony counting<br>LIVE/DEAD baclight stain	[20]
modified membrane	P. aeruginosa ATCCPAO1	Y	colony counting<br>LIVE/DEAD baclight stain<br>counting fluorescent cells	[21]
graphene oxide	E. coli BW26437		colony counting<br>SEM	[22]
modified membrane	E. coli (unspecified/unique sample type)<br>S. aureus (unspecified/unique sample type)		colony counting<br>colony counting<br>measuring flux	[23]
modified membrane	E. coli (K12) MG1655	Y	counting fluorescent cells<br>colony counting	[24]
modified membrane	P. aeruginosa ATCC700829	Y	colony counting<br>SEM<br>PI<br>DAPI	[25]
modified membrane	E. coli ATCCDH5a	Y	ATP analysis<br>ATP analysis<br>TOC	[26]
composite membranes	S. aureus ATCC6538P<br>E. coli ATCC8739		colony counting<br>LIVE/DEAD baclight stain	[27]
graphene oxide	E. coli (unspecified/unique sample type)<br>B. subtilis (unspecified/unique sample type)	Y	SEM<br>measuring flux<br>measuring flux	[28]
composite membranes	E. coli DSM 4230	Y	colony counting	[29]
composite membranes	E. coli ATCC25922			[30]
graphene oxide	E. coli ATCC35695	Y	colony counting<br>LIVE/DEAD baclight stain	[31]

graphene oxide	E. coli (unspecified/unique sample type)	Y	colony counting	[32]
graphene oxide	E. coli BW26437		colony counting	[33]
composite membranes	P. aeruginosa (unspecified/unique sample type)<br>E. coli (unspecified/unique sample type)	Y	measuring flux<br>LIVE/DEAD baclight stain	[34]
composite membranes	B. subtilis (unspecified/unique sample type)		colony counting<br>SEM	[35]
composite membranes	P. aeruginosa ATCCPAO1		colony counting<br>LIVE/DEAD baclight stain	[36]
graphene oxide<br>graphene oxide	E. coli (K12) MG1655<br>E. coli ATCC25922	Y	colony counting<br>colony counting<br>LIVE/DEAD baclight stain	[37]
composite membranes	E. coli (K12) MG1655<br>A. parasiticus JFS<br>B. pumilus LDS33	Y	colony counting	[38]
composite membranes	E. coli CCUG3274		crystal violet	[39]
composite membranes	E. coli (unspecified/unique sample type)	Y	FESEM<br>syto 9	[40]
composite membranes	S. aureus (unspecified/unique sample type)<br>E. coli (unspecified/unique sample type)		measuring flux	[41]
composite membranes	E. coli DSM1103		colony counting	[42]
composite membranes	S. aureus (unspecified/unique sample type)<br>E. coli (unspecified/unique sample type)	Y	colony counting	[43]
composite membranes	E. coli (unspecified/unique sample type)<br>P. aeruginosa (unspecified/unique sample type)		FESEM	[44]
composite membranes	P. fluorescens	Y	LIVE/DEAD baclight stain	[45]
composite membranes	S. aureus (unspecified/unique sample type)<br>P. aeruginosa (unspecified/unique sample type)<br>E. coli (unspecified/unique sample type)	Y	LIVE/DEAD baclight stain	[46]

composite membranes	P. aeruginosa ATCCPAO1		LIVE/DEAD baclight stain	[47]
composite membranes	E. coli ATCC25404<br>P. mendocina KR1		colony counting<br>DAPI<br>colony counting	[48]
composite membranes	E. coli (unspecified/unique sample type)		counting fluorescent cells	[49]
composite membranes	B. subtilis (unspecified/unique sample type)<br>B. subtilis (unspecified/unique sample type)<br>E. coli (unspecified/unique sample type)<br>E. coli (unspecified/unique sample type)		colony counting	[50]
	P. aeruginosa KCTC2004		measuring flux	[51]
composite membranes	E. coli (unspecified/unique sample type)<br>S. aureus (unspecified/unique sample type)<br>E. coli (unspecified/unique sample type)	`	LIVE/DEAD baclight stain<br>measuring flux	[52]
composite membranes	E. coli (unspecified/unique sample type)	Y	colony counting	[53]
composite membranes	P. aeruginosa (unspecified/unique sample type)		LIVE/DEAD baclight stain	[54]
composite membranes	E. coli (unspecified/unique sample type)<br>S. aureus (unspecified/unique sample type)		colony counting	[55]
composite membranes	P. aeruginosa (unspecified/unique sample type)		colony counting<br>LIVE/DEAD baclight stain	[56]
composite membranes	E. coli (unspecified/unique sample type)		LIVE/DEAD baclight stain	[57]
composite membranes	E. coli (unspecified/unique sample type)<br>S. aureus (unspecified/unique sample type)<br>P. aeruginosa (unspecified/unique sample type)	Y	colony counting<br>LIVE/DEAD baclight stain	[58]

	P. aeruginosa (unspecified/unique sample type)			
composite membranes	E. coli (unspecified/unique sample type)<br>B. subtilis (unspecified/unique sample type)		measuring flux<br>measuring flux	[59]
composite membranes	E. coli (unspecified/unique sample type)<br>S. maltophilia		LIVE/DEAD baclight stain	[60]
composite membranes	E. coli ATCC25254		colony counting<br>LIVE/DEAD baclight stain	[61]
	E. coli (unspecified/unique sample type)<br>P. fluorescens		colony counting	[62]
composite membranes	P. aeruginosa (unspecified/unique sample type)		colony counting<br>SEM<br>LIVE/DEAD baclight stain	[63]
composite membranes	S. paucimobilis		LIVE/DEAD baclight stain<br>LIVE/DEAD baclight stain	[64]
composite membranes	E. coli (unspecified/unique sample type)	Y	colony counting<br>measuring flux	[65]
composite membranes	S. aureus (unspecified/unique sample type)	Y	colony counting	[66]
composite membranes	E. coli (K12) MG1655		colony counting<br>SEM	[67]
composite membranes	E. coli (unspecified/unique sample type)<br>S. aureus (unspecified/unique sample type)	Y	colony counting<br>LIVE/DEAD baclight stain	[68]
composite membranes	E. coli (unspecified/unique sample type)<br>P. aeruginosa (unspecified/unique sample type)		SEM	[69]
composite membranes	E. coli (unspecified/unique sample type)<br>P. putida<br>P. putida		colony counting<br>LIVE/DEAD baclight stain	[70]
composite membranes	P. aeruginosa ATCCPAO1		colony counting	[71]

			LIVE/DEAD baclight stain	
composite membranes	P. putida<br><br>B. subtilis (unspecified/unique sample type)	Y	LIVE/DEAD baclight stain	[72]
composite membranes	E. coli (unspecified/unique sample type)		colony counting	[73]
composite membranes	P. putida	Y	colony counting<br>measuring flux	[74]
composite membranes	S. paucimobilis		LIVE/DEAD baclight stain<br>measuring flux	[75]
composite membranes	E. coli (unspecified/unique sample type)<br>Saccharomycetes		FESEM	[76]
	P. aeruginosa ATCCPAO1	Y	colony counting<br>LIVE/DEAD baclight stain	[77]
composite membranes	P. fluorescens			[78]
	K. oxytoca		colony counting<br>LIVE/DEAD baclight stain	[79]
composite membranes	P. fluorescens<br>S. wittichii RW1		counting fluorescent cells<br>LIVE/DEAD baclight stain<br>counting fluorescent cells	[80]
composite membranes	E. coli ATCC25922<br>S. aureus ATCC6538P<br>S. aureus ATCC6538P		OD<br>LIVE/DEAD baclight stain	[81]
composite membranes	S. aureus (unspecified/unique sample type)<br>E. coli (unspecified/unique sample type)		colony counting<br>FESEM	[82]
composite membranes	P. fluorescens		counting fluorescent cells<br>LIVE/DEAD baclight stain<br>measuring flux	[83]
composite membranes	E. coli (unspecified/unique sample type)		colony counting	[84]
composite membranes	P. fluorescens<br>P. putida		counting fluorescent cells	[85]
composite membranes	E. coli (unspecified/unique sample type)<br>B. subtilis (unspecified/unique sample type)			[86]
composite membranes	P. putida<br>P. fluorescens			[87]
composite membranes	E. coli MTCC1302	Y	SEM<br>LIVE/DEAD baclight stain	[88]
graphene oxide	S. aureus ATCC1901		colony counting	[89]

	E. coli ATCC8739<br><br>E. coli ATCC8739		colony counting<br>colony counting	
composite membranes	E. coli BW26437		LIVE/DEAD baclight stain	[90]
composite membranes	E. coli (K12) MG1655		counting fluorescent cells	[91]
graphene oxide	E. coli (unspecified/unique sample type)		colony counting	[92]
composite membranes	E. coli (unspecified/unique sample type)		LIVE/DEAD baclight stain	[93]
composite membranes	E. coli ATCC47,076<br>P. aeruginosa KCTC2004<br>S. aureus KCTC 3881		colony counting<br>LIVE/DEAD baclight stain	[94]
composite membranes	P. fluorescens<br><br>S. epidermidis ATCC12228		LIVE/DEAD baclight stain<br>SEM	[95]
graphene oxide	E. coli (K12) ATCC700926		colony counting<br>OD	[96]
composite membranes	E. coli (unspecified/unique sample type)		colony counting	[97]
composite membranes	S. marcescens		measuring flux<br>OD	[98]
composite membranes	E. coli (unspecified/unique sample type)		colony counting<br><br><br>SEM	[99]
composite membranes	B. subtilis ATCC6633<br>E. coli ATCC8739<br><br><br>C. testosteroni I2	 <br><br><br>Y	colony counting<br>LIVE/DEAD baclight stain<br>SEM	[100]
composite membranes	E. coli ACIB 8277<br>S. aureus ATCC6538P	Y	colony counting<br>SEM	[101]
composite membranes	E. coli (K12) MG1655		colony counting	[102]
composite membranes	E. coli (unspecified/unique sample type)		OD	[103]
composite membranes	S. aureus (unspecified/unique sample type)<br>P. aeruginosa (unspecified/unique sample type)			[104]
composite membranes	E. coli DH5a<br>S. aureus CICC10201	Y	colony counting	[105]
composite membranes	E. coli (unspecified/unique sample type)	Y	SEM	[106]
composite membranes	P. aeruginosa (unspecified/unique sample type)	Y	measuring flux	[107]
composite membranes	E. coli BW26437<br>P. aeruginosa ATCC26437<br>S. aureus ATCC8325	Y	colony counting<br>LIVE/DEAD baclight stain	[108]

	P. aeruginosa ATCC26437			
composite membranes	E. coli (K12) KCTC1116<br>S. aureus KCTC1928	Y	SEM	[109]
composite membranes	P. aeruginosa ATCC27853	Y	SEM	[110]
composite membranes	E. coli DH5a		colony counting<br>SEM<br>LIVE/DEAD baclight stain<br>measuring flux	[111]
composite membranes	P. aeruginosa ATCC700829	Y	Dapi	[112]
composite membranes	E. coli (K12) MG1655		LIVE/DEAD baclight stain	[113]
composite membranes	E. coli (unspecified/unique sample type)<br>B. subtilis (unspecified/unique sample type)		colony counting	[114]
composite membranes	E. coli (unspecified/unique sample type)<br>B. subtilis (unspecified/unique sample type)		colony counting<br>measuring flux	[115]
composite membranes	B. subtilis ATCC27370<br>E. coli ATCC10798	Y	colony counting	[116]
	E. coli BW26437	Y	colony counting	[117]
composite membranes	P. aeruginosa NCIM 2036<br>S. aureus NCIM 5345	Y	colony counting<br>SEM<br>measuring flux	[118]
composite membranes	P. aeruginosa ATCCPAO1	Y	LIVE/DEAD baclight stain	[119]
composite membranes	E. coli (unspecified/unique sample type)<br>P. aeruginosa (unspecified/unique sample type)<br>S. aureus (unspecified/unique sample type)<br>Enterococcus faecalis (unspecified/unique sample type)	Y	OD<br>LIVE/DEAD baclight stain	[120]
composite membranes	E. coli (unspecified/unique sample type)		measuring flux	[121]
graphene oxide	E. coli (unspecified/unique sample type)	Y	LIVE/DEAD baclight stain	[122]

composite membranes	E. coli (unspecified/unique sample type)	Y	SEM<br>colony counting	[123]
composite membranes	E. coli (unspecified/unique sample type)<br>S. aureus (unspecified/unique sample type)		colony counting<br>LIVE/DEAD baclight stain<br>SEM	[124]
composite membranes	E. coli ATCC8739<br>S. aureus ATCC6538P	Y	colony counting	[125]
composite membranes	P. aeruginosa ATCCPAO1	Y	colony counting<br>SEM<br>LIVE/DEAD baclight stain	[126]
composite membranes	E. coli (unspecified/unique sample type)	Y	colony counting<br>SEM<br>counting fluorescent cells	[127]
composite membranes	E. coli DH5a		SEM	
composite membranes	E. coli ATCC25922<br>S. aureus ATCC255923	Y	colony counting<br>SEM<br>counting fluorescent cells	
composite membranes	E. coli ATCCDH5a		colony counting	
composite membranes	E. coli (unspecified/unique sample type)<br>B. subtilis (unspecified/unique sample type)		colony counting	
composite membranes	E. coli (unspecified/unique sample type)		colony counting	
composite membranes	E. coli Nissle 1917	Y	measuring flux	
	P. aeruginosa P60	Y	LIVE/DEAD baclight stain<br>colony counting	
	P. aeruginosa ATCCPAO1	Y	colony counting<br>LIVE/DEAD baclight stain<br>OD<br>measuring flux	

Table S2. Semi-systematic review raw data answering: Which organisms are found in biofilms on RO membranes?

**Membrane Setup**	**Water Type**	**Identification Method**	**Bacterial Identity**	**Source**
Moving Bed Biofilm Reactor inoculated with activated sludge	WWTP influent	16S rRNA	Acinetobacter<br>Methyloversatilis<br>Denitratisoma<br>Lactobacillus<br>Pseudomonas<br>unidentified_Spirochaetaceae<br>Methylotenera	[128]

			Sulfuritalea<br>Sutterella<br>Streptococcus	
RO membrane from pilot plant	/	16S rRNA	Deltaproteobacteria<br>Bacilli<br>Betaproteobacteria<br>Sphingobacteria<br>Flavobacteria<br>Alphaproteobacteria<br>Gammaproteobacteria	[129]
benchscale RO	Seawater sample	16S rRNA	Blastocatella fastidiosa (JQ309130)<br>Nocardia cyriacigeorgica (BAFY01000107)<br>Lysinimonas soli (JN378395)<br>Propionibacterium acnes (AB042288)<br>Luteibaculum oceani (KC169812)<br>Spongiibacterium flavum (FJ348473)<br>Blastopirellula marina (AANZ01000021)<br>Planctomyces maris (ABCE01000043)<br>Sphaeronema italicum (AY428765)<br>Kordiimonas aquimaris (GU289640)<br>Methlyoligella solikamskensis (JQ773444)<br>Ochrobactrum oryzae (AM041247)<br>Rhodoplanes elegans (D25311)<br>Tepidamorphus gemmatus (GU187912)<br>Labrenzia aggregate (AUUW01000037)<br>Magnetospira thiphila (EU861390)<br>Nisaea denitrificans (DQ665838)<br>Tistlia consotensis (EU728658)<br>Caedibacter acanthamoebae (AF132138)<br>Seathiella chungangensis (KF482756)<br>Limnobacter thiooxidans (AJ289885)<br>Acidovorax temperans (AF078766)<br>Acidovorax caeni (AM084006)<br>Marinobacter algicola (ABCP01000031)<br>Maricoccus atlantica (KC997601)<br>Porticoccus hydrocarbonoclasticus (JN088732)<br>Endoriftia Persephone (AFOC01000137)	[130]

			Thiohalomonas nitratireducens (DQ836238)<br>Deulfuromonas svalbardensis (AY835388)<br>Pedosphaera parvula (ABOX01000003)<br>Juniperus virginiana (AF131092)<br>Pinus thunbergii (D17510)<br>Thermoanaerobaculum aquaticum (JX420244)<br>Flagellimonas eckloniae (DQ191180)<br>Fabibacter pacificus (KC005305)<br>Nitrospina gracilis (L35504)<br>Phycisphaera mikurensis (AP012338)<br>Scalindua sorokinii (AY257181)<br>Maritalea porphyrae (AB583774)<br>Methyloceanibacter caenitepidi (AB794104)<br>Labrenzia alexandrii (ACCU01000015)<br>Hyphomonas oceanitis (AF082797)<br>Roseovaruis mucosus (AJ534215)<br>Phaeobacter caeruleus (AM943630)<br>Micavibrio aeruginosavorus (CP002382)<br>Pelagibius litoralis (DQ401091)<br>Magnetospira thiophila (EU861390)<br>Microvirga subterranean (FR733708)<br>Roseovarius lutimaris (JF714703)<br>Marinicauda pacifica (JQ045549)<br>Polyangium brachysporum (AM410613)<br>Legionella dresdenensis (AM747393)<br>Theoalkalibirbrio thiocyanodenitrificans (AY360060)<br>Alcanivorax balearicus (AY686709)<br>Microbulbifer gwangyangensis (JF751045)<br>Oceanibaculum pacificum (FJ463255)<br>Methylophaga marina (X95459)<br>Pelobacter carbinolicus (CP001734)<br>Deulfohalobium retbaense (CP001734)<br>Roseibacillus ishigakijimensis (AB331888)<br>Roseibacillus ponti (AB331889)	
RO plant samples	/	16S rRNA	Acidovorax<br>Flavobacterium	[131]

			Mycobacterium<br>Tatlockia<br>Aminobacter<br>Hyphomicrobium<br>Pedobacter<br>Bacillus<br>Leptothrix<br>Rhodobacter<br>Devosia<br>Methylobacterium<br>Sphingopyxis	
Benchscale NF, RO, GDM	Shale gas wastewater	16S rRNA	Nitrosomonas<br>Denitromonas<br>Azoarcus<br>Uncl._o__Oligosphaerales<br>Psedoxanthomonas<br>Methylobacterium<br>Hyphomicrobium<br>Dialister<br>Alkalibacter<br>Pseudomonas<br>Pelagibacterium<br>Uncl._d__Bacteria<br>Planktosalinus<br>Uncl._o__Sphingomonadales<br>Uncl._f__Erythrobacteraeae<br>Uncl._o__Bradymonadales<br>Sphingopyxis<br>C1-B045_f__Porticoccaceae<br>Legionella<br>Acetobacterium<br>Muricauda<br>Sphingosinicella<br>Pusillimonas<br>Labrenzia<br>Citeitalea<br>Paracoccus<br>Glycocaulis<br>PAUC26f_o__Solibacterales<br>Chromatocurvus<br>Iodidimonas<br>Rubrimonas<br>Thalassobaculum<br>Lacimicrobium<br>Uncl._f__Phycissphaeraceae<br>Oceanibacterium<br>Dichotomicrobium<br>Tistlia<br>Uncl._f__Cryomorphaceae<br>Uncl._c__Deltaproteobacteria<br>Arenibacter<br>Magnetospira<br>Methyloceanibacter	[132]

			Uncl._p__BRC1<br>Porphyrobacter<br>Uncl._p__Hydrogenedentes<br>Uncl._f__Rhodospirillaceae<br>Alcanivorax<br>Uncl._o__Rickettsiales<br>Uncl._c__Gemmatimonadetes<br>Rhodovulum<br>Uncl._f__Halieaceae<br>Methylophaga<br>Roseavarius<br>Pseudohongeilla<br>Bryobacter<br>Filomicrobium<br>Uncl._p__Proteobacteria<br>Marinobacter<br>Uncl._c__Gammaproteobacteria<br>Unc._f__Rhodobactaceae<br>Uncl._c__Alphaproteobacteria<br>Uncl._Rhizobiales<br>Uncl._p__Bacteroidetes<br>Rehaibacterium<br>SM1A02_f__Phycisphaeraceae	
lab & pilotscale GDM	Seawater sample next to desalination plant	16S rRNA	Hydrogenedentes<br>Firmicutes<br>Candidatus<br>Saccharibacteria<br>Verrucomicrobia<br>Proteobacteria<br>Planctomycetes<br>Chloroflexi<br>Chlamydiae<br>Bacteroidetes<br>Actinobacteria	[133]
benchscale RO	MBR effluent from WWTP	16S rRNA	env.OPS 17<br>Xanthomonadaceae<br>Sphingomonadaceae<br>Rickettsiales Incertae Sedis<br>Rhizobiaceae<br>P3OB-42<br>Moraxellaceae<br>Methylophilaceae<br>Hyaloperonospora arabidopsidis<br>Helicobacteraceae<br>Halothiobacillaceae<br>Flavobacteriaceae<br>Cytophagaceae<br>Comamonadaceae<br>Chitinophagaceae<br>Burkholderiaceae<br>Bradyrhizobiaceae<br>Blastocatellaceae<br>Anaerolineaceae	[134]

RO samples from PSDP	Seawater sample etc.	16S rRNA, phenotypes	Ochrobactrum<br>Agrobacterium<br>Sphingobium<br>Sphingomonas<br>Sphingopyxis<br>Achromobacter<br>Burkholderia<br>Pandoraea<br>Ralstonia<br>Delftia<br>Shewanella<br>Acinetobacter<br>Pseudomonas<br>Cytophaga<br>Flavobacterium<br>Pedobacter<br>Sphingobacterium<br>Rhodococcus<br>Microbacterium<br>Cellulomonas<br>Alicyclobacillus<br>Bacillus<br>Streptococcus	[135]
RO membrane from SWRO plant	Seawater sample	MAG analysis	Candidatus Omnitrophica<br>Chlamydiae<br>Acidobacteria<br>Nitrospirae<br>Gemmatimonadetes<br>Verrucomicrobia<br>Planctomycetes<br>Cyanobacteria<br>Chloroflexi<br>Firmicutes<br>Actinobacteria<br>Bacteriodetes<br>Proteobacteria	[136]
Pilot scale RO	MBR effluent	16S rRNA	Pedobacter composti<br>Pseudomonas pseudoalcaligenes<br>Pseudomonas anguilliseptica<br>Herbaspirillum huttiense<br>Limnobacter thiooxidans<br>Pseudomonas veronii<br>Azoarcus tolulyticus<br>Reyranella massiliensis<br>Hydrogenophaga pseudoflava<br>Hyphomicrobium vulgare<br>Gemmobacter lanyuensis<br>Pseudomonas stutzeri<br>Pseudomonas caeni<br>Brevundimonas denitrificans<br>Pseudomonas mendocina<br>Falvobacterium cucumis<br>Gemmobacter megaterium	[137]

			Brevundimonas bullata<br>Subtercola frigoramans<br>Sphingobium fontiphilum<br>Sphingpbium xenophagum	
Sample membranes	Sea, brackish, well water	16S rRNA, ITS(fungi)	Acidobacteria<br>Acidimicrobiales<br>Actinomycetales<br>Solirubrobacterales<br>Cytophagales<br>Flavobacteriales<br>Saprospirales<br>Chlamydiales<br>Chlorobi<br>Cyanobacteria<br>Bacillales<br>Nitrospirales<br>Acetothermales<br>Planctomycetes<br>Phycisphaerales<br>Planctomycetales<br>Alphaproteobacteria<br>Caulobacterales<br>Kiloniellales<br>Kordiimonadales<br>Rhizobiales<br>Rhodobacterales<br>Rhodospirillales<br>Sphingomonadales<br>Burkholderiales<br>Rhodocyclales<br>Myxococcales<br>Deltaproteobacteria<br>Syntrophobacterales<br>Gammaproteobacteria<br>Legionellales<br>Oceanospirllales<br>Thiotrichales<br>Xanthomonadales	[138]
RO, MF	Influent and effluent of MF-RO system	16S rRNA	Comamonadaceae<br>Sphingomonadaceae<br>Oxalobacteraceae<br>Planktophila_f<br>Flavobacteriaceae<br>SAR11-4_f<br>Chitinophagaceae<br>Mycobacteriaceae<br>Sphingobacteriaceae<br>Moraxellaceae<br>Lautropia_f<br>Rhodobacteraceae<br>Alcaligenaceae	[139]

RO, UF	Influent and effluent of UF-RO system	16S rRNA	Starkeya<br>Acidovorax<br>Luteimonas<br>Pelomonas<br>Xanthobacter<br>Hydrogenophaga<br>Sphingopyxis<br>Pseudoxanthomonas<br>Bdellovibrio<br>Bosea<br>Afipia<br>Sediminibacterium<br>Gemmobacter<br>Roseomonas<br>Bacteriovorax<br>Devosia<br>Thermomonas<br>Leifsonia<br>Comamonas<br>Flacovacterium<br>Methyloversatilis<br>Hyalangium<br>Saccharibacteria<br>Shinella<br>Brevundimonas<br>Chitinophaga<br>Pseudomonas<br>Citrobacter<br>Legionella<br>Herminiimonas<br>Variovorax<br>Parachlamydia<br>Fluviicola<br>Ohtaekwangia<br>Pedobacter<br>Ferruginibacter<br>Nereida<br>Sphingomonas<br>Emticicia<br>Roseateles<br>Salinibacterium<br>Hyphomicrobium<br>Clostridium sensu stricto<br>Mesorhizobium<br>Adhaeribacter<br>Cytophaga<br>Cloacibacterium<br>Phaselicystis	[140]
RO pilot system	Wastewater from oil refinery	16S rRNA, culture	Leifsonia<br>Microbacterium<br>Acidocella<br>Ancylobacter<br>Bosea	[141]

			Bradyrhizobium<br>Breundimonas<br>Devosia<br>Ensifer<br>Ferrovibrio<br>Hirschia<br>Kaistia<br>Labrys<br>Magnetospirillum<br>Mesorhizobium<br>Novosphingobium<br>Parvularcula<br>Pedomicrobium<br>Rhizobium<br>Rhizomicrobium<br>Rhodobacter<br>Shinella<br>Sphingobium,<br>Sphingopyxis<br>Woodsholea<br>Acidovorax<br>Burkholderia<br>Cupreavidus<br>Limnobacter<br>Methylibium<br>Sulfuritale<br>Zoogloea<br>Blastocatella<br>Runella<br>Bdellovibrio<br>Vampirovibrio<br>Flavobacterium<br>Alkanindiges<br>Aquicella<br>Escherichia<br>Shigella<br>Pseudomonas<br>Rheinheimera<br>Thioprofundum<br>Thiothrix<br>Nitrospira<br>Coraliomargarita<br>Opitutus<br>Sediminibacterium<br>Brevifollis	
RO plant	Wastewater from WWTP	16S rRNA, flow cytometry, HPC, ATP measurements	Rhodobacter<br>Caldilinea<br>Phyllobacteriaceae<br>Stenotrophomonas<br>Longilinea<br>Phycisphaera<br>Massilia<br>Opitutus	[142]

			Nitrosopumilus<br>Haliangiumcaldilineaceae<br>Sinobacteraceae<br>Nannocystineae<br>Hymenobacter<br>Rhodovulum<br>Anaerolineaceae<br>Owenweeksia<br>Spirochaeta<br>Barnesiella<br>Legionella<br>Hgci_clade<br>Cystbacterineaeop3 (candidate division)<br>Saprospiraceae<br>Planctomycetaceae<br>Candidatus_chloracidobacterium<br>Psuedomonas<br>Rhodospirillaceae	
Labscale NF	Wastewater effluent	High throughput illumina pyrosequencing	Betaprotobacteria<br>Gammaproteobacteria<br>Unclassified Proteobacteria<br>Alphaporteobacteria<br>Actinobacteria<br>Verrucomicrobia<br>Chloroflexi<br>Minor phylum<br>Deltaproteobacteria<br>Nitrospira<br>Bacteroidetes<br>Acidobacteria<br>Epsilonproteobacteria<br>Unclassified Phylum<br>Cyanobacteria<br>Planctomycetes<br>Firmicutes<br>TM7<br>Deinococcus-Thermus<br>Chlamydiae<br>Synergistetes<br>Gemmatimonadetes	[143]
AWPF (advanced water purification facility)	WWTP influent	Shotgun (WGS) sequencing	Acidovorax<br>Acinetobacter<br>Aeromonas<br>Afipia<br>Arcobacter<br>Bacteroides<br>Bifidobacterium<br>Caulobacter<br>Chryseobacterium<br>Comamonas<br>Elizabethkingia<br>Flavobacterium	[144]

			Hydrogenophaga<br>Klebsiella<br>Limnohabitans<br>Mycobacterium<br>Pseudomonas<br>Rhodocyclaceae<br>Sediminibacterium<br>Sphingobium<br>Sphingopyxis<br>Thauera<br>Thiobacillus	
/	WWTP influent	16S rRNA	Thauera<br>Denitratisoma<br>Propionivibrio<br>Sphingobium<br>Terrimonas<br>Nitrosomonas<br>Pseudomonas<br>Ornatilinea<br>Anaerolinae<br>Planctomyces<br>Filimonas<br>Streptococcus<br>Clostridium<br>Ignavibacterium<br>Comamonas<br>Arcobactor<br>Nitrospira<br>Sphingomonas<br>Microvirgula<br>Bacteroides<br>Undibacterium<br>Mycobacterium<br>Cloacibacterium<br>Chryseobacterium	[145]
DMF, (PA)RO with BER	Raw seawater	ATP analysis, 16S rRNA 454 pyrosequencing	Dehalococcoides<br>Trichodesmium<br>Nitrospira<br>Sphingomonas<br>Hyphomonas<br>Sneathiella<br>Erythrobacter<br>Maricaulis<br>Roseobacter<br>Thiohalobacter<br>Phaeobacter<br>Oceanicola<br>Thalassospira<br>Alteromonas<br>Marinobacter<br>Algisphaera<br>Micavibrio<br>Cyanobacterium	[146]

RO sample from WWTP	Synthetic water inoculated with activated sludge	16S rRNA PCR	Strenotrophomonas maltophilia strain MHF ENV20<br>Bacillus cereus strain ZL-1<br>Bacillus sp. Sd-16<br>Bacillus cereus strain 2<br>Delftia tsuruhatensis strain BN-HKY6<br>Pseudomonas sp. SJT25	[147]
UF, NF, RO spiral wound membranes from dairy processing plants	Pastuerized milk, condensed water, UF whey permeate, Bleached cheese whey	16S rRNA (enzymatic pretreatment and a phenol-chloroform extraction)	Other Bacilli<br>Other Firmicutes<br>Alphaproteobacteria<br>Methylobacterium<br>Other Alphaproteobacteria<br>Betaproteobacteria<br>Burkholderia<br>Petrobacter<br>Other Betaproteobacteria<br>Gammaproteobacteria<br>Acinetobacter<br>Citrobacter<br>Cronobacter<br>Klebsiella<br>Pseudoalteromonas<br>Psychrobacter<br>Other Gammaproteobacteria<br>Other Proteobacteria	[148]
Labscale RO	Suplemented TWW solution	16S rRNA (PCR amplification and Illumina sequencing)	Aeromonadaceae<br>Alphaproteobacteria<br>Bacteroidetes<br>Bradyrhizobiaceae<br>Burkholderiaceae<br>Caulobacteaceae<br>Chitinophagaceae<br>Chromatiaceae<br>Comamonadaceae<br>Cystobacteraceae<br>Cytophagaceae	[149]
Pilotscale SWRO	Treated feed water (Chlorine and sand filtration)	16S rRNA	Marinobacter adhaerens<br>Vibrio atlanticus<br>Ruegeria atlantica<br>Muricauda lutimaris<br>Parasphingopyxis lamellibrachiae<br>Erythrobacter longus<br>Roseovarius albus<br>Rhodanobacter ginsengisoli<br>Pelagibius litoralis<br>Maribacter polysiphoniae<br>Alcanivorax jadensis<br>Hyphomonas chukchienses<br>Parvularcula lutaonensis<br>Sphingopyxis litoris<br>Methylophaga thiooxydans<br>Bacillus subtiliis	[150]

/	Feed water and permeate water from RO drinking plant	16S rRNA, MALDI-TOF MS	Ensifer adhaerens<br>Stenotrophomonas<br>Serratia<br>Rhizobium<br>Pseudomonas<br>Enterobacter<br>Acinetobacter<br>Acidovorax<br>Achromobacter<br>Stenotrophomonas Maltophilia<br>Ilyobacter Delafieldii<br>Pseudomonas veronii<br>Pseudomonas libanensis<br>Pseudomonas jessenii<br>Pseudomonas frederiksbergensis<br>Pseudomonas koreensis<br>Pseudomonas putida<br>Pseudomonas nitroreducens<br>Pseudomonas proteolytica<br>Pseudomonas mandelii	[151]
MF membrane samples from wastewater treatment plant		16S rRNA, culturing	Enterobacteriaceae<br>Moraxellaceae<br>Pseudomonadaceae<br>Comamonadaceae<br>Xanthomonadaceae<br>Phyllobacteriaceae<br>Bacillaceae<br>Paenibacillaceae<br>Microbacteriaceae	[152]
Labscale forward osmosis system	Supplemented annamox effluent	16S rRNA Illumina sequencing	Prosthecobacter<br>Dokdonella<br>Dok59<br>Kucncnia<br>Candidatus Jettenia<br>Candidatus Brocadia<br>Sediminibacterium	[153]
KAUST desalination plant	Raw seawater<br>Brackish water RO permeate<br>Drinking water (chlorinated)<br>RO influent<br>RO permeate<br>Spruce media permeate	16S rRNA pyrosequencing, PCR	Sphingomonadales<br>Parvularculales<br>Rhodobacterales<br>Rhodospirillales<br>Caulobacterales<br>Rhizobiales<br>Rickettsiales<br>Rhodocyclales<br>Nitrosomonadales<br>Methylophilales<br>Burkholderiales<br>Hydrogenophilales<br>Alteromonadales<br>Thiotrichales<br>Pseudomonadales<br>Methylococcales<br>Oceanospirillales	[154]

			Enterobacteriales<br>Chromatiales<br>Legionellales<br>Aeromonadales<br>Desulfobacterales<br>Desulfarculales<br>Syntrophobacterales<br>Bdellovibrionales<br>Myxococcales<br>Desulfuromonadales<br>Bacilli<br>Ws3<br>Synergistia<br>Oscillatoriophycideae<br>Verrucomicrobiae<br>Lentisphaeria<br>Nitrospira<br>Planctomycea<br>Caldilineae<br>Bacteroidia<br>Phycisphaerae<br>Opitutae<br>Candidatus_Thiobios<br>Anaerolineae<br>Cytophagia<br>Clostridia<br>Planctomycetacia<br>Sphingobacteria<br>Synechococcophycideae<br>Flavobacteria	
ADOM desalination facility	Raw seawater<br>Pre RSF water<br>RSF anthracite layer (biofilm)<br>RSF sand layer (biofilm)<br>Post RSF water<br>CF membrane<br>Post CF water<br>RO membrane modules (biofilm)<br>Brine<br>Product	16S rRNA pyrosequencing	Actinobacteria<br>Alphaproteobacteria<br>Bacteroidetes<br>Betaproteobacteria<br>Chloroflexi<br>Deltaproteobacteria<br>Acidobacteria<br>Epsilonproteobacteria<br>Planctomycetes<br>Gammaproteobacteria<br>Rhizobiales<br>Rhodobacterales<br>SAR11 cluster<br>Nitrosomonadales<br>Deltaproteobacteria<br>Chromatiales<br>Legionellales<br>Oceanospirillales<br>Xanthomonadales	[155]
SWRO module	Raw seawater<br>DMF inlet<br>DMF outlet	16S rRNA pyrosequencing	Cyanobacteria<br>Proteobacteria<br>Gammaproteobacteria<br>Alphaproteobacteria	[156]

	SWRO inlet<br>SWRO permeate<br>SWRO brine		Bacillales<br>Rhodobacteraceae<br>Rhodospirillaceae<br>Hyphomonadaceae<br>Pseudomonas<br>Janthinobacterium<br>Kordiimonas<br>Legionella<br>Flavobacterium<br>Methylophilus<br>Ralstonia<br>Corynebacterium<br>Burkholderia<br>Streptococcus<br>Prevotella<br>Methylobacterium<br>Silicibacter<br>Staphylococcus<br>Acidocella<br>Pedobacter	
Labscale RO	Water inoculated with bacteria from industrial MBR and RO	454 pyrosequencing	Proteobacter<br>Bacteroidetes<br>Betaproteobacter<br>Gammaproteobacteria<br>Alphaproteobacteria<br>Sphingobacteriia<br>Chitinophagaceae<br>Oxalobacteraceae<br>Enterobacteriaceae<br>Comamonadaceae<br>Sphingobacteriaceae<br>Aeromonadaceae<br>Flavobacteriaceae	[157]
RO plant	Secondary WWTP effluent<br>RO influent<br>RO effluent<br>Brackish water RO permeate<br>Drinking water (chlorinated)<br>RO influent<br>RO permeate<br>Spruce media permeate	16S rRNA pyrosequencing	Acidobacteria<br>Sphingobacteria<br>Bacteroidetes<br>Firmicutes<br>Fusobacteria<br>Alphaproteobacteria<br>Betaproteobacteria<br>Deltaproteobacteria<br>Epsilonproteobacteria<br>Gammaproteobacteria	[158]
Labscale RO	UF permeate from HG-MBR	16S rRNA	Proteobacteria<br>Acidobacteria<br>Actinobacteria<br>Bacteroidetes<br>Sphingomonadales<br>Rhizobiales<br>Burkholderiales<br>Xanthomonadales	[159]

			Pseudomonadales<br>Sphingobacteriales	
Labscale RO	Raw seawater	16S rRNA pyrosequencing	Alphaproteobacteria<br>Rhodobacteraceae bacterium<br>Roseobacter<br>Sulfitobacter<br>Phaeobacter<br>Antarcticicola litoralis<br>Betaproteobacteria<br>Gammaproteobacteria<br>Cycloclasticus<br>Colwellia<br>Spongiibacter<br>Pseudoalteromonas<br>Marinobacter<br>Pseudomonas<br>Deltaproteobacteria<br>Firmicutes<br>Bacteroidetes<br>Actinobacteria<br>Loktanella<br>Rhodovulum<br>Pseudoruegeria<br>Thalassobilus<br>Sagittula,<br>Sphingopyxis<br>Erythrobacter<br>Jannaschia<br>Rhizobium<br>Sphingobium<br>Neptunomonas<br>Moraxellaceae<br>Glaciecola<br>Shewanella<br>Oceanospirillaceae bacterium<br>Psychrobacter<br>Alcanivorax<br>Thiohalomonas<br>Serratia<br>Microbulbifer<br>Cellvibrio<br>Cycloclasticus sp. Phe42 (GQ345342)<br>Colwellia sp. BSw20968 (GU166136)<br>Spongiibacter sp. JAMGA14 (AB526337)<br>Pseudoalteromonas sp. B149 (FN295744)<br>Marinobacter sp. YKS2 (AB504895)<br>Pseudomonas stutzeri BBSPN3 (GU594474)	[160]

			Pseudidiomarina sp. KYW314 (FJ768737)	
Labscale RO	Raw seawater	16S rRNA pyrosequencing<br>ATP analysis	Actinobacteria<br>Cyanobacteria<br>Firmicutes<br>Lentisphaerae<br>Planctomycetes<br>Betaproteobacteria<br>Epsilonproteobacteria<br>Bacteroidetes<br>Deinococcus-Thermus<br>Fibrobacteres<br>OD1<br>Verrucomicrobia<br>Alphaproteobacteria<br>Deltaproteobacteria<br>Gammaproteobacteria<br>Antarctobacter<br>Citreicella<br>Pelagibaca<br>Rhodobacter<br>Rhodobacteraceae<br>Roseobacter<br>Roseovarius nubinhibens<br>Sulfitobacter<br>Methylophaga<br>Pseudidiomarina homiensis	[161]
RO pilot plant	WWTP secondary effluent<br>Activated sludge	16S rRNA	Afipia felis<br>Bradyrhizobium<br>Bradyrhizobium yuanmingense<br>Ensifer<br>Ensifer sinorhizobium Saheli<br>Rhizobium etli<br>Roseomonas<br>Sinorhizobium<br>Sphingomonadales<br>Sphingomonas<br>Sphingopyxis panaciterrae<br>Aquamonas fontana<br>Aquamonas<br>Methylibium aquaticum<br>Nitrosomonadaceae	[162]

			Pelomonas<br>Zoogloea<br>Helicobacteraeae<br>Legionellaceae<br>Methylococcaceae<br>Methylococcales<br>Methylothermus<br>Pseudomonadaceae<br>Pseudomonas entomophila<br>Stentrophomonas acidaminiphila<br>Gammaproteobacteria<br>Kineosporiaceae<br>Kouleothrix<br>Microbacteriaceae<br>Mycobacterium cosmeticum<br>Mycobacterium nonchromogenicum<br>Tetrasphaera<br>Flavobacteriaceae<br>Caldilinea<br>Lactococcus lactis<br>Anabaena<br>Tm7	
Fouled SWRO membranes	Raw seawater<br>Pretreated seawater	16S rRNA<br>ATP analysis	Proteobacteria<br>Bacteroidetes	[163]
CF and RO samples from commercial desalination	/	Total Genomic DNA analysis<br>16S rDNA	Aciditerrimonas ferrireducens IC-180<br>Winogradskyella sp. poriferorum UST030701-295<br>Bacillus algicola strain LS7<br>Bacillus zhanjiangensis JSM 099021<br>Bacillus cohnii DSM 6307<br>Bacillus vietnamensis 15-1<br>Bacillus decolorationis LMG 19507<br>Blastopirellula marina DSM 3645<br>Zavarzinella formosa A10<br>Mesorhizobium albiziae CCBAU 61158<br>Parvularcula lutaonensis CC-MMS-1<br>Leisingera aquimarina CCUG<br>Pseudoruegeria lutimaris HD-43<br>Ruegeria lacuscaerulensis ITI-1157<br>Nautella italica CCUG 55857<br>Thalassobius aestuarii JC2049<br>Rhodobacter veldkampii ATCC35703<br>Parvularcula bermudensis HTCC2503<br>Rickettsia montanensis ATCC VR-611	[164]

	Maribius salinus CL-SP27<br>Paracoccus fistulariae KCTC 22803<br>Phaeobacter daeponensis TF-218<br>Phaeobacter caeruleus CCUG 55859<br>Loktanella pyoseonensis JJM85T<br>Pseudidiomarina taiwanensis strain PIT1<br>Kangiella koreensis DSM 16069<br>Kangiella aquimarina SW-154<br>Kangiella spongicola A79<br>Shigella flexneri ATCC 29903<br>Legionella brunensis 441-1<br>Legionella beliardensis strain Montbeliard A1<br>Thioalkalivibrio denitrificans ALJD<br>Thioalkalivibrio thiocyanodenitrificans ARhD1<br>Vibrio diabolicus HE800<br>Photobacterium rosenbergii LMG 22223<br>Desulfuromusa ferrireducens 102<br>Bdellovibrio bacteriovorus HD100<br>Geobacter bemidjiensis Bem<br>Geobacter bremensis Dfr1<br>Roseibacillus ishigakijimensis MN1-741<br>Candidatus Solibacter usitatus Ellin6076<br>Caldithrix palaeochoryensis MC<br>Thermonema lapsum ATCC 43542<br>Adhaeribacter aquaticus MBRG1.5<br>Ekhidna lutea BiosLi/39<br>Wandonia haliotis Haldis-1<br>Meridianimaribacter flavus NH57N<br>Bizionia echini KMM 6177<br>Owenweeksia hongkongensis UST20020801<br>Flavobacterium haoranii LQY-7<br>Candidatus Aquirestis calciphila MS-Falk1-L<br>Solitalea koreensis R2A36-4<br>Leptolinea tardivitalis YMTK-2<br>Bellilinea caldifistulae GOMI-1<br>Caldilinea aerophila STL-6-O1<br>Fusibacter paucivorans SEBR 4211<br>Thermaerobacter composti Ni80<br>Sporacetigenium mesophilum ZLJ115<br>Geosporobacter subterraneus VNs68

			Caldicoprobacter oshimai JW/HY-331<br>Clostridium sp. pascui DSM 10365<br>Nitrospira marina Nb-295<br>Blastopirellula marina DSM 3645<br>Gemmata obscuriglobus UQM 2246<br>Blastopirellula marina DSM 3645<br>Zavarzinella formosa A10<br>Phycisphaera mikurensis FYK2301M01<br>Parvularcula bermudensis HTCC2503<br>Jhaorihella thermophila CC-MHSW-1<br>Ruegeria marina ZH17<br>Pseudoruegeria lutimaris HD-43<br>Afifella pfennigii AR2102<br>Erythrobacter flavus SW-46<br>Limnobacter thiooxidans CS-K2<br>Thauera terpenica 58Eu<br>Malikia spinosa ATCC 14606<br>Azoarcus indigens VB32<br>Curvibacter delicatus LMG 4328<br>Balneatrix alpaca 4-87<br>Shigella sonnei GTC 781<br>Shigella flexneri ATCC 29903<br>Cellvibrio mixtus subsp. Mixtus ACM 2603<br>Marinicella litoralis KMM 3900<br>Thermomonas haemolytica A50-7-3<br>Thiohalocapsa marina JA142<br>Legionella gresilensis ATCC 700509<br>Natronocella acetinitrilica strain ANL 6-2<br>Haliangium tepidum SMP-10<br>Geobacter metallireducens GS-15<br>Hippea maritima MH2	
Elsa purification plant<br>RO membrane sample	River water	16S rRNA	Cryobacterium psychrotolerans, DQ515963<br>Leifsonia kafniensis, AM889135<br>Clone ROM_78, HE575376<br>Clone ROM_17, HE575377<br>Leifsonia rubra, AJ459101<br>Salinibacterium amurskyense, AF539697<br>Clone ROM_5, HE575378<br>Clone ROM_96, HE575379<br>Frigoribacterium mesophilum, EF466126<br>Frigoribacterium faeni, AM410686	[165]

Frondihabitans australicus,
DQ525859
Clone ROM_93, HE575382
Clone ROM_2, HE575380
Microcella alkaliphile, AJ717385
Microcella putealis, AJ717388
Yonghaparkia alkaliphile,
DQ256087
Clavibacter michiganensis subsp.
M, U09762
Clone ROM_92, HE575381
Propionicicella superfundia,
DQ176646
Propionicimonas paludicola,
AB078858
Micropruina glycogenica,
AB012607
Clone ROM_77, HE575383
Nocardioides fonticola, EF626689
Nocardioides pyridinolyticus,
U61298
Nocardioides terrigena, EF363712
Nocadrioides halotolerans,
EF466122
Actinomadura chokoriensis,
AB3311730
Clone ROM_18, HE575385
Actinomadura bangladeshensis,
AB331652
Actinomadura livida, AF163116
Actinomadura yumaensis,
AF163122
Actinomadura meyerae, AY273787
Clone ROM_1, HE575384
Actinomadura cremea subsp.
Cremea, AF134067
Actinomadura glauciflava,
AB184612
Actinomadura formosensis,
AJ293703
Actinomadura napierensis,
AY568292
Clone ROM_13, HE575386
Uncultured bacterium, FJ671519
Sphaerobacter thermophilus,
AJ420142
Thermomicrobium roseum,
M34115
Clone ROM_28, HE575388
Canthobacter flavus, X94199
Xanthobacter aminoxidans,
AF399969
Clone ROM_3, HE575387

			Xanthobacter agilis, X94198<br>Clone ROM_25, HE575389<br>Phenylobacterium conjuctum, AJ227767<br>Clone ROM_23, HE575390<br>Sphingomonas terrae, D13727<br>Sphingomonas adhaesiva, D13727<br>Sphingopyxis ginsengisoli, AB245343<br>Sphingomonas macrogoltabidus, D13723<br>Sphingopyxis witflariensis, AJ416410<br>Clone ROM_20, HE575391<br>Thiobacillus sajanensis, DQ390445<br>Thiobacillus denitrificans, AJ243144<br>Clostridium estertheticum subsp. 1, AJ506115<br>Clostridium frigoris, AJ506117<br>Clostridium bowmanii, AJ506119<br>Clostridium tagluense, DQ296031<br>Clone ROM_87, HE575394<br>Clone ROM_15, HE575393<br>Clostridium peptidivorans, AF156796<br>Clostridium tetanomorphum, DQ241819<br>Clostridium aminovalericum, X73436<br>Clostridium jejuense, AY494606<br>Clone ROM_10, HE575392<br>Clostridium populeti, X71853<br>Clostridium phytofermentans, CP000885<br>Clone ROM_59, HE575395	
Biofouled SWRO membrane<br><br>Cartridge Filter	Raw seawater	16S rRNA	Donghicola eburneus strain SW-277 (DQ667965)<br>Uncultured bacterium clone S25_436 (EF574092)<br>Loktanella sp.K4B-4 (FJ889559)<br>Uncultured Rhodobacteraceae bacterium clone NdSurf79 (FJ753141)<br>Thalassobius sp. (FJ889559)<br>Marine sponge bacterium FILTER4C220m (EU34644)<br>Roseobacter sp. (AY258102)<br>Nautella sp. (FJ161344)<br>Ruegeria sp. (FJ357642)<br>Rhodobacteraceae bacterium (FM163068)<br>Arctic bacterium NP26 (EU196330)	[166]

			Robiginitomaculum sp. G5 (FJ230838)<br>Novosphingomonas sp. (AB070237)<br>Sphingomonadaceae bacterium ACEMC 2-1 (FM07237)<br>Marine gamma proteobacterium Fun-110 (DQ107393)<br>Leucothrix muco (X87277)<br>Isolate B1<br>Shewanella sp. (EF105395)<br>Isolate B4<br>Isolate B6<br>Vibrio sp.1A8 (EU854873)<br>Isolate B3<br>Alteromonas sp. (FJ652055)<br>Isolate B2<br>Uncultured planctomycete clone Hal 25 (AM422930)<br>Uncultured Chloroflexi bacterium clone GoM IDB-09(EU735030)<br>Lewinella cohaerens (AB301614)<br>Uncultured bacterium clone SGUS1259 (FJ202110)<br>Cellulaphaga sp. (AB180390)<br>Isolate B5<br>Flavobacteria bacterium SOMBO 59 (AJ936938)<br>Lacinutrix sp. (DQ530481)<br>Uncultured Flavobacteria bacterium (AM 279207)<br>Winogradskyella sp. (EU727254)<br>Flavobacteriaceae bacterium ALC1 (EF527870)	
pilot scale SWRO plant	Raw seawater<br>Permeates	16S rRNA	Aestuariibacter halophilus<br>Aestuariibacter litoralis<br>Alteromonas hispanica<br>Alcanivorax dieselolei<br>Alcanivorax balearicus<br>Alcanivorax hongdengensis<br>Alteromonas addita<br>Alteromonas stellipolaris<br>Alteromonas macleodii<br>Alteromonas marina<br>Glaciecola mesophila<br>Colwellia aestuarii<br>Colwellia polaris<br>Colwellia piezophila<br>Glaciecola chathamensis<br>Glaciecola agarilytica<br>Glaciecola polaris<br>Glaciecola pallidula	[167]

Umboniibacter marinipuniceus
Saccharophagus degradans
Microbulbifer salipaludis
Arcobacter marinus
Arcobacter nitrofigilis
Arcobacter halophilus
Sulfurovum lithotrophicum
Nitratifractor salsuginis
Sulfuricurvum kujiense
Owenweeksia hongkongensis
Wandonia haliotis
Kordia periserrulae
Marinovum algicola
Thalassobius aestuarii
Oceanicola pacificus
Nautella italica
Oceanibulbus indolifex
Shimia marina
Parvularcula bermudensis
Parvularcula lutaonensis
Parvibaculum indicum
Ponticoccus litoralis
Sulfitobacter dubius
Pseudoruegeria aquimaris
Dinoroseobacter shibae
Glaciecola lipolytica
Colwellia asteriadis
Glaciecola punicea
Aestuariibacter salexigens
Marinobacterium marisflavi
Marinobacterium lutimaris
Neptuniibacter caesariensis
Enhygromyxa salina
Plesiocystis pacifica
Nannocystis exedens
Anaeromyxobacter dehalogenans
Geobacter uraniireducens
Phaselicystis flava
Candidatus Arcobacter sulfidicus
Kordiimonas lacus
Kordiimonas gwangyangensis
Devosia geojensis
Altererythrobacter marinus
Roseovarius mucosus
Roseovarius tolerans
Roseovarius halotolerans
Oceanibaculum pacificum
Nisaea nitritireducens
Oceanibaculum indicum
Sneathiella glossodoripedis
Sneathiella chinensis
Devosia subaequoris
Marinicella litoralis

			Kistimonas asteriae<br>Endozoicomonas montiporae<br>Alteromonas litorea<br>Alteromonas genovensis<br>Thalassomonas actiniarum<br>Thalassomonas viridans<br>Thalassomonas haliotis<br>Granulosicoccus coccoides<br>Granulosicoccus antarcticus<br>Marinobacter lutaoensis	
	Seawater<br>Brackish water<br>Wastewater<br>Freshwater<br>Activated sludge-treated waste effluent	16S rRNA<br>Culturing	Simonsiella sp.<br>Bosea sp.<br>Rhizobium sp.<br>Proteobacteria<br>Cytophaga–Flexibacter–Bacteroides group<br><br>Firmicutes<br>Sphingomonas sp.<br>Bacteroides<br>Actinobacteria<br>Rhizobiales<br>Dermacoccus sp.<br>Microbacterium sp.<br>Rhodopsedumonas sp.<br>Bradyrhizobium<br>Bosea<br>Planctomycetes<br>Acidobacter<br>Pseudomonas spp.<br>Corynebacterium<br>Pseudomonas<br>Bacillus<br>Arthrobacter<br>Flavobacterium<br>Aeromonas<br>Penicillium<br>Trichoderma<br>Mucor<br>Acinetobacter<br>Lactobacillus<br>Alcaligenes<br>Moraxella<br>Micrococcus<br>Serratia	[168]
full scale desalination plant	open intake water; RO feed water after conventional pre-treatment	PCR= 16S rRNA tree	Alphaproteobacteria<br>Defluvicoccus AACY020273010<br>Hyphomicrobiaceae DQ431901<br>Parvularcula FJ516787<br>Parvularcula EU236361	[169]

Phyllobacteriaceae EU236398
Rhizobiales GQ348477
Rhodobacteraceae FJ716871
Roseovarius EF471647
marine group AEGEAN-169
EF471704
marine group AEGEAN-169
AACY023897748
Rhodospirillaceae GQ264068
SAR116 EU799440
SAR116 AY664095
SAR11clade DQ009166
SAR11clade EU805335
Rhodospirillales EU237396
SAR11clade EP879548
OCS 116 clade AB106120
OCS 116 clade EU236400
Micavibrio FJ202882
Rhodobium_2 FJ745192
Rickettsiales EF 516885
Rickettsiales EU804393
Betaproteobacteria
Ideonella AB240317
Deltaproteobacteria
Nannocystineae EU283371
SAR324clade EF574189
Gammaproteobacteria
Rheinheimera EF076757
Ectothiorhodospiraceae GQ246404
SAR86 clade AACY020555669
SAR86 clade FJ745006
SAR86 clade EF574960
SAR86 clade FJ745145
SAR86 clade EF575172
Actinobacteria
Acidimicrobineae EP609371
Bacteroidetes
Bruminmicrobium AF507867
Cryomorphaceae EU183317
Flavobacteriaceae EU799420
Flavobacterium EF573073
Flavobacteriaceae EF572094
Polaribacter AY794064
Bizionia EU143366
Flavobacteriaceae FJ545454
Flavobacterium GQ988780
Flexibacteraceae AF406540
Haliscomenobacter EF644787
BD1-5
BD1-5 FJ203485
Chloroflexi
Caldilineacea AB250571
Anaerolineae EU050928

			Deferribacteres<br>SAR406clade AACY023373955<br>Planctomycetes<br>Planctomyces DQ811897<br>Planctomyces DQ395893<br>Planctomycetaceae AY 592313<br>Planctomyces FJ664808<br>Planctomyces DQ811897<br>Planctomycetaceae FJ 202841<br>Verrucomicrobia<br>Verrucomicrobiales DQ300578<br>Incertae_sedis FJ478940	
spiral-wound RO mems from RO unit	seawater	PCR via recombinant cells= 16S rRNA gene	Salipiger sp. PTG4-12<br>Pelagibaca sp. F6<br>Thalassobius aestuarii Tf-212<br>Rhodobacteraceae bacterium F9<br>Uncultured a-proteobacterium (AF473929)<br>Leisingera aquamarina LMG 24366<br>Ruegeria atlantica SS-05<br>Nautella italica LMG 24365<br>Nautella italica R-28753<br>Oceanicola granulosus HTCC2516<br>Roseobacter sp. SPO804<br>Uncultured Sulfitobacter sp. (AY697912)<br>Sulfitobacter mediterraneus (Y17387)<br>Sphinogomonas sp. JQ1-2<br>Uncultured Novosphingobium sp.<br>Parvularcula sp. CC-MMS-1<br>Uncultured Pseudorhodobacter sp. (DQ917873)<br>Alcanivorax sp. Mho1<br>Pseudoxanthomonas sp. P2-3<br>Pseudomonas borepolis<br>Alicyclobacillus pohliae CIP 109385<br>Bacillus sp. Eur1 9.5<br>Uncultured actinobacterium clone (DQ070787)<br>Uncultured high G+C gram-positive bacterium (AJ241005)<br>Candidatus Microthrix Calida TND2-4 (DQ147284)<br>Uncultured Planctomyces sp. Clone (AB189347)<br>Planctomyces sp. Schlesner 664<br>Lewinella nigricans<br>Uncultured CFB group bacterium clone (AF406541)	[170]

			Uncultured Cytophagales bacterium (AF355051)<br>Uncultured Flavobacterium sp. (AM259763)<br>Fouled RO membrane 39 alphaproteobacteria<br>sewater intake 31 alphaproteobacteria<br>seewater intake 68 alphaproteobacteria<br>sewater intake 12 alphaproteobacteria<br>sewater intake 9 alphaproteobacteria<br>Fouled RO membrane L9 alphaproteobacteria<br>Fouled RO membrane 30 alphaproteobacteria<br>Fouled RO membrane 20 alphaproteobacteria<br>Fouled RO membrane 14 alphaproteobacteria<br>Fouled RO membrane L45 alphaproteobacteria<br>Fouled RO membrane L6 gammaproteobacteria<br>Sewater intake 43 ammaproteobacteria<br>Sewater intake 58 ammaproteobacteria<br>Fouled RO membrane 48 Firmicute<br>Fouled RO membrane 3 Firmicute<br>Sewater intake 2 Actinobacter<br>Fouled RO membrane L14 Actinobacter<br>Fouled RO membrane L40 Planctomycetes<br>Fouled RO membrane L34 Bacteriodetes<br>Fouled RO membrane 01 Bacteriodetes<br>Seawater intake 55 Bacteriodetes<br>Seawater intake 52 Bacteriodetes	
NF-polyamide tubular mem; 200Da MWCO	tertiary quality wastewater effluents; synthetic media mimicking intermediate quality effluents	DGGE analysis; PCR	uncultured rape rhizosphere bacterium<br>uncultured bacterium AY053480<br>uncultured eubacterium AY038612	[171]

uncultured Bacteroidetes AJ583191
CFB group bacterium
metal-contaminated soil bacterium
uncultured bacterium AY212682
uncultured actinobacterium
UBA534677
blackwater bioreactor bacterium
BW AF394172
uncultured alphabacterium
AB193878
uncultured deltabacterium
AY218599
uncultured deltabacterium
AF414588
uncultured gammabacterium
AF418944
uncultured Ralstonia sp. Beta
bacterium
uncultured betabacterium
AY444992
gammaproteobacterium A40-1

Chitinophaga pinensis
Flavobacterium ferrugineum
Dipareforma spartinacae
Flavobacterium columnare
Curtobacterium sp.
Microbacterium keratanolyticum
Microbacterium laevaniformans
Bacillus silvestris
Brevibacillus sp. PLC-3
Bacillus sp. CPB 6
Bacillus sphaericus
Bacillus sp. ARI 3
Bacillus fusiformis
Bacillus aquamarinus
Sphingomonas sp. SKJH -30
Sphingomonas paucimobilis
Sphingomonas subarctica
Sphingomonas sp. C28242
Sphingomonas capsulata
Cystobacter fuscus
Myxococcus xanthus
Pseudomonas putida
Pseudomonas sp. NZ 024
Pseudomonas marginalis
Pseudomonas veronii
Pseudomonas sp. AEBL 3
Pseudomonas sp.
Pseudomonas sp. AY66343
Pseudomonas pavonaceae
Pseudomonas anguilliseptica

			Legionella pneumophila<br>Rolstonia sp. AY177368<br>Rolstonia sp. AY 177364<br>Rolstonia sp. FRA 01<br>Rolstonia sp.<br>Rickettsiella grylli<br>Ralstonia oxalatica<br>Ralstonia paucula<br>Wautersia sp.<br>Ralstonia taiwanensis<br>Ralstonia eutropha<br>Burkholderia anthina<br>Delftia sp.<br>Burkholderia cepacia<br>Burholderia sp.<br>Ralstonia pickettii<br>Delftia acidovorans<br>Delftia tsuruhalensis<br>Acidovorax avenae<br>Acidovorax delafieldii<br>Acidovorax sp.<br>Hydrogenophaga palleronii<br>Hydrogenophaga pseudoflava<br>Hydrogenophaga flava<br>Rhodanobacter lindanoclasticus<br>Frateuria aurantia<br>Dyella japonica	
MF, Spiral-Wound; full scale membrane process for water purification	secondary effluent (domestic WWTP or waterworks)	DNA extraction=16S rDNA clone library; FISH	Paracoccus/Rhodobacter sp.<br>Hyphomicrobium sp.<br>Azospirillum sp.<br>Xanthobacter sp.<br>Bosea sp.<br>Environmental isolate<br>Environmental clones<br>Thermomonas haemolvtica<br>Mycobacterium nonchromogenicum<br>Ctophagales sp.<br>Bacteroidetes sp.<br>Environmental clones<br>Flavobacterium sp.<br>Planctomycetes sp.<br>Nostocoida limicola<br>Bradyrhizobium sp.<br>Zoogloea sp.<br>Rhizobium sp.<br>Caulobacter sp.<br>Mesorhizobium sp.<br>Agrobacterium sp.<br>Bordetella hinzii	[172]

			Stenotrophomonas acidaminiphila<br>Nevski ramose<br>Brevibacterium sp.<br>Gordonia sp.<br>Aureobacterium sp.<br>Bacillus sp.<br>Staphylococcus sp.<br>Flavobacterium ferrugineum<br>Afipia genosp<br>Rhodopseudomonas palustris<br>Magnetospirillum sp.<br>Methylocystis parvus<br>Rhodocyclus tenuis<br>Dechlormonas agitaus<br>Tiobacillus Q<br>Legionella sainthelensi<br>Holophaga foetida<br>Geothrix fermentans<br>Sphingomonas sp.<br>Rhodospeudomonas sp.<br>Dermacoccus sp.<br>Microbacterium sp.<br>Bacillus sp.	
MBR-RO (GE Osmotics), SE-MF (full-scale hollow fiber MF mem-domestic WW), PW-RO (full-scale, spiral wound RO mem-potable water)	lab-scale MBR for wastewater + RO concentrate	T-RFLP (nitrate reductase); Bacteria-specific primers= 16S rRNA gene clone library	Oligotropha carboxidovorans S28<br>RO clone RO229<br>RO clone RO160<br>Rhodopseudomonas sp. TUT3631<br>Rhodospeudomonas palustris TUT3620<br>Bosea sp. BMA-4<br>PW-RO isolate RO3<br>RO clone RO161b<br>SE-MF isolate MF18<br>Bosea thiooxidans BI-42<br>RO clone RO215<br>Methylocella silvestris BL2<br>RO clone RO154<br>Alphaproteobacterium CRIB-02<br>Uncultured bacterium clone 661238<br>RO clone RO53<br>Alphaproteobacterium Shinshu-th1<br>RO isolate ROi16<br>Xanthobacter tagetidis TagT2C<br>Se-MF isolate MF22<br>Uncultured bacterium clone aab54f12	[173]

Mesorhizobium genosp. AA isolate Cs6145
RO isolate ROi51
Shinella zoogloeoides ATCC 19623
Gram-negative bacterium isolate DM1
Sinorhizobium meliloti Rm1021
Ochrobactrum sp. CGL-X
Brucella sp. YBJA-1
RO clone RO233
RO isolate ROi52
RO clone RO238
Uncultured Ochrobactrum sp. Clone p3
Ochrobactrum anthropi CCUG 44770
RO isolate ROi15
Ochrobactrum anthropi CCUG 44770
Ochrobactrum sp. B2
RO isolate ROi43
Sphingomonas sp. JQ1-3
Uncultured bacterium clone KRA30+14
Hydrogenophagaatypica BSB 41.8
RO clone RO219
Uncultured betaproteobacterium clone ccslm2112
RO clone RO118
Uncultured bacterium clone TSAI28
Hydrogenophaga intermedia S1
RO isolate ROi28
Denitrobacter sp. BBTR53
Castellaniella defragrans TJ4
RO isolate ROi27
Thermomonas brevis LMG 21746T
RO isolate ROi19
Thermomonas haemolytica A50-7-3
RO isolate ROi7
Stenotrophomonas maltophilia isolate FLX
RO isolate ROi55
RO clone RO156
Stenotrophomonas acidaminiphila AMX19
RO isolate ROi44
Pseudoxanthomonas kaohsiungensis J36
RO clone RO127
RO isolate ROi22
Uncultured bacterium clone SX3-79
Chimaereicella alkaliphila AC74

RO clone RO224
RO clone RO74
cf. Bergeyella CCUG 46293
Uncultured bacterium clone SS-54
RO clone RO157
Uncultured soil clone M26_Pitesti
Uncultured candidate division
TM7 bacterium clone
RO clone RO230
Uncultured bacterium clone 54
Uncultured division TM6
bacterium clone NOS7.2WL
RO isolate ROi31
Microbacterium aurum DSM 8600
RO clone RO 28
Isosphaera-like str. CJugI1
Plactomycete str. 563
Aquifex pyrophilus Kol5a

References:

1. Samantaray PK, Baloda S, Madras G, Bose S. Interlocked Dithi-Magnetospheres–Decorated MoS 2 Nanosheets as Molecular Sieves and Traps for Heavy Metal Ions. *Adv Sustain Syst*. 2019;3(6):1800153. doi:10.1002/adsu.201800153
2. Cihanoğlu A, Altinkaya SA. A facile route to the preparation of antibacterial polysulfone-sulfonated polyethersulfone ultrafiltration membranes using a cationic surfactant cetyltrimethylammonium bromide. *J Memb Sci*. 2020;594. doi:10.1016/j.memsci.2019.117438
3. Wang J, Gao X, Yu H, Wang Q, Ma Z, Li Z, Zhang Y, Gao C. Accessing of graphene oxide (GO) nanofiltration membranes for microbial and fouling resistance. *Sep Purif Technol*. 2019;215(January):91-101. doi:10.1016/j.seppur.2019.01.018
4. Moir CJ. Inhibition, injury, and inactivation of four psychrotrophic foodborne bacteria by preservatives methyl p-hydroxybenzoate and potassium sorbate. *J Food Prot*. 1992;55(5):360-366.
5. Sabri S, Najjar A, Manawi Y, Eltai NO, Al-Thani A, Atieh MA, Kochkodan V. Antibacterial properties of polysulfone membranes blended with Arabic gum. *Membranes (Basel)*. 2019;9(2):1-16. doi:10.3390/membranes9020029
6. Zhang DY, Xiong S, Shi YS, Zhu J, Hu QL, Liu J, Wang Y. Antifouling enhancement of polyimide membrane by grafting DEDA-PS zwitterions. *Chemosphere*. 2018;198:30-39. doi:10.1016/j.chemosphere.2018.01.120
7. Shukla AK, Alam J, Ansari MA, Alhoshan M, Ali FAA. Antimicrobial and antifouling properties of versatile PPSU/carboxylated GO nanocomposite membrane against Gram-positive and Gram-negative bacteria and protein. *Environ Sci Pollut Res*. 2018;25(34):34103-34113. doi:10.1007/s11356-018-3212-7
8. Li X, Cao Y, Yu H, Kang G, Jie X, Liu Z, Yuan Q. A novel composite nanofiltration membrane prepared with PHGH and TMC by interfacial polymerization. *J Memb Sci*. 2014;466:82-91. doi:10.1016/j.memsci.2014.04.034
9. Yu L, Zhou W, Li Y, Zhou Q, Xu H, Gao B, Wang Z. Antibacterial Thin-Film Nanocomposite Membranes Incorporated with Graphene Oxide Quantum Dot-Mediated Silver Nanoparticles for Reverse Osmosis Application. *ACS Sustain Chem Eng*. 2019;7(9):8724-8734. doi:10.1021/acssuschemeng.9b00598
10. Jung Y, Alayande AB, Chae S, Kim IS. Applications of nisin for biofouling mitigation of reverse osmosis membranes. *Desalination*. 2018;429(June 2017):52-59. doi:10.1016/j.desal.2017.12.003
11. Ma W, Panecka M, Tufenkji N, Rahaman MS. Bacteriophage-based strategies for biofouling control in ultrafiltration: In situ biofouling mitigation, biocidal additives and biofilm cleanser. *J Colloid Interface Sci*. 2018;523:254-265. doi:10.1016/j.jcis.2018.03.105
12. Kim TH, Lee I, Yeon KM, Kim J. Biocatalytic membrane with acylase stabilized on intact carbon nanotubes for effective antifouling via quorum quenching. *J Memb Sci*. 2018;554(March):357-365. doi:10.1016/j.memsci.2018.03.020

13. Soleymani Lashkenari A, Hamed Mosavian MT, Peyravi M, Jahanshahi M. Biofouling mitigation of bilayer polysulfone membrane assisted by zinc oxide-polyrhodanine couple nanoparticle. *Prog Org Coatings*. 2019;129(January):147-158. doi:10.1016/j.porgcoat.2018.12.012
14. Khajouei M, Jahanshahi M, Peyravi M. Biofouling mitigation of TFC membrane by in-situ grafting of PANI/Cu couple nanoparticle. *J Taiwan Inst Chem Eng*. 2018;85:237-247. doi:10.1016/j.jtice.2018.01.027
15. Raval HD, Makwana P, Sharma S. Biofouling of polysulfone and polysulfone-graphene oxide nanocomposite membrane and foulant removal. *Mater Res Express*. 2018;5(6). doi:10.1088/2053-1591/aacc82
16. Li Y, Yang Y, Li C, Hou LA. Comparison of performance and biofouling resistance of thin-film composite forward osmosis membranes with substrate/active layer modified by graphene oxide. *RSC Adv*. 2019;9(12):6502-6509. doi:10.1039/c8ra08838a
17. Rahaman MS, Thérien-Aubin H, Ben-Sasson M, Ober CK, Nielsen M, Elimelech M. Control of biofouling on reverse osmosis polyamide membranes modified with biocidal nanoparticles and antifouling polymer brushes. *J Mater Chem B*. 2014;2(12):1724-1732. doi:10.1039/c3tb21681k
18. Lien CC, Yeh LC, Venault A, Tsai SC, Hsu CH, Dizon GV, Huang YT, Higuchi A, Chang Y. Controlling the zwitterionization degree of alternate copolymers for minimizing biofouling on PVDF membranes. *J Memb Sci*. 2018;565(February):119-130. doi:10.1016/j.memsci.2018.07.054
19. Tiraferri A, Vecitis CD, Elimelech M. Covalent binding of single-walled carbon nanotubes to polyamide membranes for antimicrobial surface properties. *ACS Appl Mater Interfaces*. 2011;3(8):2869-2877. doi:10.1021/am200536p
20. Saeki D, Nagao S, Sawada I, Ohmukai Y, Maruyama T, Matsuyama H. Development of antibacterial polyamide reverse osmosis membrane modified with a covalently immobilized enzyme. *J Memb Sci*. 2013;428:403-409. doi:10.1016/j.memsci.2012.10.038
21. Kim ES, Hwang G, Gamal El-Din M, Liu Y. Development of nanosilver and multi-walled carbon nanotubes thin-film nanocomposite membrane for enhanced water treatment. *J Memb Sci*. 2012;394-395:37-48. doi:10.1016/j.memsci.2011.11.041
22. Khajouei M, Najafi M, Jafari SA. Development of ultrafiltration membrane via in-situ grafting of nano-GO/PSF with anti-biofouling properties. *Chem Eng Res Des*. 2019;142:34-43. doi:10.1016/j.cherd.2018.11.033
23. Soleymani Lashkenrai A, Najafi M, Peyravi M, Jahanshahi M, Mosavian MTH, Amiri A, Shahavi MHn. Direct filtration procedure to attain antibacterial TFC membrane: A facile developing route of membrane surface properties and fouling resistance. *Chem Eng Res Des*. 2019;149:158-168. doi:10.1016/j.cherd.2019.07.003
24. Liu Y, Rosenfield E, Hu M, Mi B. Direct observation of bacterial deposition on and detachment from nanocomposite membranes embedded with silver nanoparticles. *Water Res*. 2013;47(9):2949-2958. doi:10.1016/j.watres.2013.03.005
25. Yu C, Wu J, Zin G, Di Luccio M, Wen D, Li Q. D-Tyrosine loaded nanocomposite membranes for environmental-friendly, long-term biofouling control. *Water Res*. 2018;130:105-114. doi:10.1016/j.watres.2017.11.037
26. Xu S, Wang P, Sun Z, Liu C, Lu D, Qi J, Ma J. Dual-functionalization of polymeric membranes via cyclodextrin-based host-guest assembly for biofouling control. *J Memb Sci*. 2019;569(October 2018):124-136. doi:10.1016/j.memsci.2018.10.012
27. Díez B, Amariei G, Rosal R. Electrospun Composite Membranes for Fouling and Biofouling Control. *Ind Eng Chem Res*. 2018;57(43):14561-14570. doi:10.1021/acs.iecr.8b04011
28. Najjar A, Sabri S, Al-Gaashani R, Kochkodan V, Atieh MA. Enhanced fouling resistance and antibacterial properties of novel graphene oxide-arabic gum polyethersulfone membranes. *Appl Sci*. 2019;9(3). doi:10.3390/app9030513
29. Li H, Cheng D, Dong L, Qian F. Enhancement in permselectivity and antibacterial performances of polyamide ro membranes via surface modification of agcl nanoparticles. *Desalin Water Treat*. 2018;116:19-28. doi:10.5004/dwt.2018.22478
30. Chen H, Huang M, Wang Z, Gao P, Cai T, Song J, Zhang Y, Meng L. Enhancing rejection performance of tetracycline resistance genes by a TiO2/AgNPs-modified nanofiber forward osmosis membrane. *Chem Eng J*. 2020;382(September 2019). doi:10.1016/j.cej.2019.123052
31. Firouzjaei MD, Shamsabadi AA, Aktij SA, Song J, Zhang Y, Meng L. Exploiting Synergetic Effects of Graphene Oxide and a Silver-Based Metal-Organic Framework to Enhance Antifouling and Anti-Biofouling Properties of Thin-Film Nanocomposite Membranes. *ACS Appl Mater Interfaces*. 2018;10(49):42967-42978.

doi:10.1021/acsami.8b12714
32. Pang J, Kang Z, Wang R, Xu B, Nie X, Fan L, Zhang F, Du X, Feng S, Sun D. Exploring the sandwich antibacterial membranes based on UiO-66/graphene oxide for forward osmosis performance. *Carbon N Y*. 2019;144:321-332. doi:10.1016/j.carbon.2018.12.050
33. Lu X, Feng X, Zhang X, Chukwu MN, Osuji CO, Elimelech M. Fabrication of a Desalination Membrane with Enhanced Microbial Resistance through Vertical Alignment of Graphene Oxide. *Environ Sci Technol Lett*. 2018;5(10):614-620. doi:10.1021/acs.estlett.8b00364
34. Seyedpour SF, Rahimpour A, Najafpour G. Facile in-situ assembly of silver-based MOFs to surface functionalization of TFC membrane: A novel approach toward long-lasting biofouling mitigation. *J Memb Sci*. 2019;573(October 2018):257-269. doi:10.1016/j.memsci.2018.12.016
35. Abdulazeez I, Matin A, Khan M, Khaled MM, Ansari MA, Akhtar S, Rehman S. Facile preparation of antiadhesive and biocidal reverse osmosis membranes using a single coating for efficient water purification. *J Memb Sci*. 2019;591(January):117299. doi:10.1016/j.memsci.2019.117299
36. Shtreimer Kandiyote N, Avisdris T, Arnusch CJ, Kasher R. Grafted Polymer Coatings Enhance Fouling Inhibition by an Antimicrobial Peptide on Reverse Osmosis Membranes. *Langmuir*. 2019;35(5):1935-1943. doi:10.1021/acs.langmuir.8b03851
37. Cheng W, Lu X, Kaneda M, Zhang W, Bernstein R, Ma J, Elimelech M. Graphene Oxide-Functionalized Membranes: The Importance of Nanosheet Surface Exposure for Biofouling Resistance. *Environ Sci Technol*. 2019. doi:10.1021/acs.est.9b05335
38. Zhang HL, Gao YB, Gai JG. Guanidinium-functionalized nanofiltration membranes integrating anti-fouling and antimicrobial effects. *J Mater Chem A*. 2018;6(15):6442-6454. doi:10.1039/c8ta00342d
39. Wibisono Y, Yandi W, Golabi M, Nugraha R, Cornelissen ER, Kemperman AJB, Ederth T, Nijmeijer K. Hydrogel-coated feed spacers in two-phase flow cleaning in spiral wound membrane elements: Anovel platform for eco-friendly biofouling mitigation. *Water Res*. 2015;71:171-186. doi:10.1016/j.watres.2014.12.030
40. Wang SY, Fang LF, Cheng L, Jeon S, Kato N, Matsuyama H. Improved antifouling properties of membranes by simple introduction of zwitterionic copolymers via electrostatic adsorption. *J Memb Sci*. 2018;564(July):672-681. doi:10.1016/j.memsci.2018.07.076
41. Seyedpour SF, Rahimpour A, Shamsabadi AA, Soroush M. Improved performance and antifouling properties of thin-film composite polyamide membranes modified with nano-sized bactericidal graphene quantum dots for forward osmosis. *Chem Eng Res Des*. 2018;139:321-334. doi:10.1016/j.cherd.2018.09.041
42. Duong PHH, Daumann K, Hong PY, Ulbricht M, Nunes SP. Interfacial Polymerization of Zwitterionic Building Blocks for High-Flux Nanofiltration Membranes. *Langmuir*. 2019;35(5):1284-1293. doi:10.1021/acs.langmuir.8b00960
43. Zhang X, Wang Z, Tang CY, Ma J, Liu M, Ping M, Chen M, Wu Z. Modification of microfiltration membranes by alkoxysilane polycondensation induced quaternary ammonium compounds grafting for biofouling mitigation. *J Memb Sci*. 2018;549(December 2017):165-172. doi:10.1016/j.memsci.2017.12.004
44. Sathish Kumar R, Arthanareeswaran G. Nano-curcumin incorporated polyethersulfone membranes for enhanced anti-biofouling in treatment of sewage plant effluent. *Mater Sci Eng C*. 2019;94(October 2017):258-269. doi:10.1016/j.msec.2018.09.010
45. Hirsch UM, Teuscher N, Rühl M, Heilmann A. Plasma-enhanced magnetron sputtering of silver nanoparticles on reverse osmosis membranes for improved antifouling properties. *Surfaces and Interfaces*. 2019;16(October 2018):1-7. doi:10.1016/j.surfin.2019.04.003
46. Zhu J, Hou J, Zhang Y, Tian M, He T, Liu J, Chen V. Polymeric antimicrobial membranes enabled by nanomaterials for water treatment. *J Memb Sci*. 2018;550(November 2017):173-197. doi:10.1016/j.memsci.2017.12.071
47. Kim HJ, Kim DG, Yoon H, Choi YS, Yoon J, Lee JC. Polyphenol/FeIII Complex Coated Membranes Having Multifunctional Properties Prepared by a One-Step Fast Assembly. *Adv Mater Interfaces*. 2015;2(14):1-8. doi:10.1002/admi.201500298
48. Zodrow K, Brunet L, Mahendra S, Li D, Zhang A, Li Q, Alvarez PJJ. Polysulfone ultrafiltration membranes impregnated with silver nanoparticles show improved biofouling resistance and virus removal. *Water Res*. 2009;43(3):715-723. doi:10.1016/j.watres.2008.11.014
49. Tian E, Wang X, Wang X, Ren Y, Zhao Y, An X. Preparation and Characterization of Thin-Film Nanocomposite Membrane with High Flux and Antibacterial Performance for Forward Osmosis. *Ind Eng Chem Res*.

2019;58(2):897-907. doi:10.1021/acs.iecr.8b04476
50. Gao Y, Zhao S, Qiao Z, Zhou Y, Song B, Wang Z, Wang J. Reverse osmosis membranes with guanidine and amine enriched surface for biofouling and organic fouling control. *Desalination*. 2018;430(June 2017):74-85. doi:10.1016/j.desal.2017.12.055
51. Choi W, Lee C, Lee D, Won YJ, Lee GW, Shin MG, Chun B, Kim TS, Park HD, Jung HW, et al. Sharkskin-mimetic desalination membranes with ultralow biofouling. *J Mater Chem A*. 2018;6(45):23034-23045. doi:10.1039/c8ta06125d
52. Rahimpour A, Seyedpour SF, Aghapour Aktij S, Dadashi Firouzjaei M, Zirehpour A, Arabi Shamsabadi A, Khoshhal Salestan S, Jabbari M, Soroush M. Simultaneous Improvement of Antimicrobial, Antifouling, and Transport Properties of Forward Osmosis Membranes with Immobilized Highly-Compatible Polyrhodanine Nanoparticles. *Environ Sci Technol*. 2018;52(9):5246-5258. doi:10.1021/acs.est.8b00804
53. Kasongo G, Steenberg C, Morris B, Kapenda G, Jacobs N, Aziz M. Surface grafting of polyvinyl alcohol (Pva) cross-linked with glutaraldehyde (ga) to improve resistance to fouling of aromatic polyamide thin film composite reverse osmosis membranes using municipal membrane bioreactor effluent. *Water Pract Technol*. 2019;14(3):614-624. doi:10.2166/wpt.2019.047
54. Kim TS, Park SH, Park D, Lee JH, Kang S. Surface immobilization of chlorhexidine on a reverse osmosis membrane for in-situ biofouling control. *J Memb Sci*. 2019;576(October 2018):17-25. doi:10.1016/j.memsci.2019.01.030
55. Pan SF, Ke XX, Wang TY, Liu Q, Zhong L Bin, Zheng YM. Synthesis of Silver Nanoparticles Embedded Electrospun PAN Nanofiber Thin-Film Composite Forward Osmosis Membrane to Enhance Performance and Antimicrobial Activity. *Ind Eng Chem Res*. 2019;58(2):984-993. doi:10.1021/acs.iecr.8b04893
56. Wen Y, Chen Y, Wu Z, Liu M, Wang Z. Thin-film nanocomposite membranes incorporated with water stable metal-organic framework CuBTTri for mitigating biofouling. *J Memb Sci*. 2019;582(April):289-297. doi:10.1016/j.memsci.2019.04.016
57. Zhang Y, Ruan H, Guo C, Liao J, Shen J, Gao C. Thin-film nanocomposite reverse osmosis membranes with enhanced antibacterial resistance by incorporating p-aminophenol-modified graphene oxide. *Sep Purif Technol*. 2020;234(September 2019):116017. doi:10.1016/j.seppur.2019.116017
58. Park SH, Hwang SO, Kim TS, Cho A, Kwon SJ, Kim KT, Park HD, Lee JH. Triclosan-immobilized polyamide thin film composite membranes with enhanced biofouling resistance. *Appl Surf Sci*. 2018;443:458-466. doi:10.1016/j.apsusc.2018.03.003
59. Wang Y, Wang Z, Wang J, Wang S. Triple antifouling strategies for reverse osmosis membrane biofouling control. *J Memb Sci*. 2018;549(August 2017):495-506. doi:10.1016/j.memsci.2017.12.047
60. Maggay IV, Yeh TH, Venault A, Hsu CH, Dizon GV, Chang Y. Tuning the molecular design of random copolymers for enhancing the biofouling mitigation of membrane materials. *J Memb Sci*. 2019;588(June):117217. doi:10.1016/j.memsci.2019.117217
61. Farid MU, Khanzada NK, An AK. Understanding fouling dynamics on functionalized CNT-based membranes: Mechanisms and reversibility. *Desalination*. 2019;456(October 2018):74-84. doi:10.1016/j.desal.2019.01.013
62. Piyadasa C, Yeager TR, Gray SR, Stewart MB, Ridgway HF, Pelekani C, Orbell JD. Antimicrobial effects of pulsed electromagnetic fields from commercially available water treatment devices-controlled studies under static flow conditions. *J Chem Technol Biotechnol*. 2017;93(3):871-877. doi:10.1002/j
63. Mao C, Mohanraj G, Kandiyote NS, Kasher R, Arnusch CJ. UV mediated attachment of short Arginine-Tryptophan antimicrobial peptides on reverse osmosis membrane surfaces inhibit Pseudomonas aeruginosa biofilm. *Desalination*. 2018;431(June 2017):73-79. doi:10.1016/j.desal.2017.12.027
64. Yang Z, Saeki D, Matsuyama H. Zwitterionic polymer modification of polyamide reverse-osmosis membranes via surface amination and atom transfer radical polymerization for anti-biofouling. *J Memb Sci*. 2018;550(October 2017):332-339. doi:10.1016/j.memsci.2018.01.001
65. Wei X, Wang Z, Chen J, Wang J, Wang S. A novel method of surface modification on thin-film-composite reverse osmosis membrane by grafting hydantoin derivative. *J Memb Sci*. 2010;346(1):152-162. doi:10.1016/j.memsci.2009.09.032
66. Liu C, Guo Y, Wei X, Wang C, Qu M, Schubert DW, Zhang C. An outstanding antichlorine and antibacterial membrane with quaternary ammonium salts of alkenes via in situ polymerization for textile wastewater treatment. *Chem Eng J*. October 2019:123306. doi:10.1016/j.cej.2019.123306
67. Yin J, Yang Y, Hu Z, Deng B. Attachment of silver nanoparticles (AgNPs) onto thin-film composite (TFC)

membranes through covalent bonding to reduce membrane biofouling. *J Memb Sci*. 2013;441:73-82. doi:10.1016/j.memsci.2013.03.060
68. Li N, Yu L, Xiao Z, Jiang C, Gao B, Wang Z. Biofouling mitigation effect of thin film nanocomposite membranes immobilized with laponite mediated metal ions. *Desalination*. 2020;473. doi:10.1016/j.desal.2019.114162
69. Huang J, Arthanareeswaran G, Zhang K. Effect of silver loaded sodium zirconium phosphate (nanoAgZ) nanoparticles incorporation on PES membrane performance. *Desalination*. 2012;285:100-107. doi:10.1016/j.desal.2011.09.040
70. Karkhanechi H, Takagi R, Ohmukai Y, Matsuyama H. Enhancing the antibiofouling performance of RO membranes using Cu(OH)2 as an antibacterial agent. *Desalination*. 2013;325:40-47. doi:10.1016/j.desal.2013.06.015
71. Alpatova A, Kim ES, Sun X, Hwang G, Liu Y, Gamal El-Din M. Fabrication of porous polymeric nanocomposite membranes with enhanced anti-fouling properties: Effect of casting composition. *J Memb Sci*. 2013;444:449-460. doi:10.1016/j.memsci.2013.05.034
72. Peng F, Hoek EMV, Damoiseaux R. High-content screening for biofilm assays. *J Biomol Screen*. 2010;15(7):748-754. doi:10.1177/1087057110374992
73. Kwak SY, Kim SH, Kim SS. Hybrid organic/inorganic reverse osmosis (RO) membrane for bactericidal anti-fouling. 1. Preparation and characterization of TiO2 nanoparticle self-assembled aromatic polyamide thin-film-composite (TFC) membrane. *Environ Sci Technol*. 2001;35(11):2388-2394. doi:10.1021/es0017099
74. Ronen A, Semiat R, Dosoretz CG. Impact of ZnO embedded feed spacer on biofilm development in membrane systems. *Water Res*. 2013;47(17):6628-6638. doi:10.1016/j.watres.2013.08.036
75. Yang Z, Saeki D, Takagi R, Matsuyama H. Improved anti-biofouling performance of polyamide reverse osmosis membranes modified with a polyampholyte with effective carboxyl anion and quaternary ammonium cation ratio. *J Memb Sci*. February 2019. doi:10.1016/j.memsci.2019.117529
76. Shen X, Zhao Y, Feng X, Bi S, Ding W, Chen L. Improved antifouling properties of PVDF membranes modified with oppositely charged copolymer. *Biofouling*. 2013;29(3):331-343. doi:10.1080/08927014.2013.772142
77. Yu J, Baek Y, Yoon H, Yoon J. New disinfectant to control biofouling of polyamide reverse osmosis membrane. *J Memb Sci*. 2013;427:30-36. doi:10.1016/j.memsci.2012.09.057
78. Lei J, Mayer C, Freger V, Ulbricht M. Synthesis and characterization of poly(ethylene glycol) methacrylate based hydrogel networks for anti-biofouling applications. *Macromol Mater Eng*. 2013;298(9):967-980. doi:10.1002/mame.201200297
79. Lutskiy MY, Avneri-Katz S, Zhu N, Itsko M, Ronen Z, Arnusch CJ, Kasher R. A microbiology-based assay for quantification of bacterial early stage biofilm formation on reverse-osmosis and nanofiltration membranes. *Sep Purif Technol*. 2015;141:214-220. doi:10.1016/j.seppur.2014.12.003
80. Weinman ST, Bass M, Pandit S, Herzberg M, Freger V, Husson SM. A switchable zwitterionic membrane surface chemistry for biofouling control. *J Memb Sci*. 2018;548(September 2017):490-501. doi:10.1016/j.memsci.2017.11.055
81. Zhang X, Ma J, Tang CY, Wang Z, Ng HY, Wu Z. Antibiofouling Polyvinylidene Fluoride Membrane Modified by Quaternary Ammonium Compound: Direct Contact-Killing versus Induced Indirect Contact-Killing. *Environ Sci Technol*. 2016;50(10):5086-5093. doi:10.1021/acs.est.6b00902
82. Qiu WZ, Zhao ZS, Du Y, Hu MX, Xu ZK. Antimicrobial membrane surfaces via efficient polyethyleneimine immobilization and cationization. *Appl Surf Sci*. 2017;426:972-979. doi:10.1016/j.apsusc.2017.07.217
83. Marré Tirado ML, Bass M, Piatkovsky M, Ulbricht M, Herzberg M, Freger V. Assessing biofouling resistance of a polyamide reverse osmosis membrane surface-modified with a zwitterionic polymer. *J Memb Sci*. 2016;520:490-498. doi:10.1016/j.memsci.2016.07.027
84. Bodner EJ, Kandiyote NS, Lutskiy MY, Albada HB, Metzler-Nolte N, Uhl W, Kasher R, Arnusch CJ. Attachment of antimicrobial peptides to reverse osmosis membranes by Cu(i)-catalyzed 1,3-dipolar alkyne-azide cycloaddition. *RSC Adv*. 2016;6(94):91815-91823. doi:10.1039/c6ra21930f
85. Semião AJC, Habimana O, Casey E. Bacterial adhesion onto nanofiltration and reverse osmosis membranes: Effect of permeate flux. *Water Res*. 2014;63:296-305. doi:10.1016/j.watres.2014.06.031
86. Wang H, Zhou Y, Wang Y, Wang Z, Wang J. Biguanidine functional chitooligosaccharide modified reverse osmosis membrane with improved anti-biofouling property. *RSC Adv*. 2018;8(73):41938-41949. doi:10.1039/c8ra09291e
87. Habimana O, Casey E. Biofilm recruitment under nanofiltration conditions: the influence of resident biofilm

structural parameters on planktonic cell invasion. *Microb Biotechnol*. 2018;11(1):264-267. doi:10.1111/1751-7915.12881
88. Biswas P, Bandyopadhyaya R. Biofouling prevention using silver nanoparticle impregnated polyethersulfone (PES) membrane: E. coli cell-killing in a continuous cross-flow membrane module. *J Colloid Interface Sci*. 2017;491:13-26. doi:10.1016/j.jcis.2016.11.060
89. de Faria AF, de Moraes ACM, Andrade PF, da Silva DS, do Carmo Gonçalves M, Alves OL. Cellulose acetate membrane embedded with graphene oxide-silver nanocomposites and its ability to suppress microbial proliferation. *Cellulose*. 2017;24(2):781-796. doi:10.1007/s10570-016-1140-6
90. Ye G, Lee J, Perreault F, Elimelech M. Controlled Architecture of Dual-Functional Block Copolymer Brushes on Thin-Film Composite Membranes for Integrated "defending" and "attacking" Strategies against Biofouling. *ACS Appl Mater Interfaces*. 2015;7(41):23069-23079. doi:10.1021/acsami.5b06647
91. Ma W, Rahaman MS, Therien-Aubin H. Controlling biofouling of reverse osmosis membranes through surface modification via grafting patterned polymer brushes. *J Water Reuse Desalin*. 2015;5(3):326-334. doi:10.2166/wrd.2015.114
92. Ma W, Soroush A, Luong TVA, Rahaman MS. Cysteamine- and graphene oxide-mediated copper nanoparticle decoration on reverse osmosis membrane for enhanced anti-microbial performance. *J Colloid Interface Sci*. 2017;501:330-340. doi:10.1016/j.jcis.2017.04.069
93. Hibbs MR, McGrath LK, Kang S, Adout A, Altman SJ, Elimelech M, Cornelius CJ. Designing a biocidal reverse osmosis membrane coating: Synthesis and biofouling properties. *Desalination*. 2016;380:52-59. doi:10.1016/j.desal.2015.11.017
94. Park SH, Kim SH, Park SJ, Ryoo S, Woo K, Lee JS, Kim TS, Park HD, Park H, Park YI, et al. Direct incorporation of silver nanoparticles onto thin-film composite membranes via arc plasma deposition for enhanced antibacterial and permeation performance. *J Memb Sci*. 2016;513:226-235. doi:10.1016/j.memsci.2016.04.013
95. Choudhari S, Habimana O, Hannon J, Allen A, Cummins E, Casey E. Dynamics of silver elution from functionalised antimicrobial nanofiltration membranes. *Biofouling*. 2017;33(6):520-529. doi:10.1080/08927014.2017.1331436
96. Jiang Y, Wang WN, Liu D, Nie Y, Li W, Wu J, Zhang F, Biswas P, Fortner JD. Engineered crumpled graphene oxide nanocomposite membrane assemblies for advanced water treatment processes. *Environ Sci Technol*. 2015;49(11):6846-6854. doi:10.1021/acs.est.5b00904
97. Wang J, Gao X, Wang Q, Sun H, Wang X, Gao C. Enhanced biofouling resistance of polyethersulfone membrane surface modified with capsaicin derivative and itaconic acid. *Appl Surf Sci*. 2015;356:467-474. doi:10.1016/j.apsusc.2015.08.095
98. Yamanouchi S, Nasuno E, Ohno M, Okano C, Iimura K, Okuda T, Nishijima W, Kato N. Enhancement effects of cationic contaminants from bacteria on cake layer formation and biofouling on an RO membrane. *Biotechnol Bioprocess Eng*. 2017;22(3):281-286. doi:10.1007/s12257-017-0093-4
99. Wang J, Sun H, Gao X, Gao C. Enhancing antibiofouling performance of Polysulfone (PSf) membrane by photo-grafting of capsaicin derivative and acrylic acid. *Appl Surf Sci*. 2014;317:210-219. doi:10.1016/j.apsusc.2014.08.102
100. Liu X, Foo LX, Li Y, Lee JY, Cao B, Tang CY. Fabrication and characterization of nanocomposite pressure retarded osmosis (PRO) membranes with excellent anti-biofouling property and enhanced water permeability. *Desalination*. 2016;389:137-148. doi:10.1016/j.desal.2016.01.037
101. Al Aani S, Gomez V, Wright CJ, Hilal N. Fabrication of antibacterial mixed matrix nanocomposite membranes using hybrid nanostructure of silver coated multi-walled carbon nanotubes. *Chem Eng J*. 2017;326:721-736. doi:10.1016/j.cej.2017.06.029
102. Zhang Y, Wan Y, Shi Y, Pan G, Yan H, Xu J, Guo M, Qin L, Liu Y. Facile modification of thin-film composite nanofiltration membrane with silver nanoparticles for anti-biofouling. *J Polym Res*. 2016;23(5):1-9. doi:10.1007/s10965-016-0992-7
103. Falath W, Sabir A, Jacob KI. Highly improved reverse osmosis performance of novel PVA/DGEBA cross-linked membranes by incorporation of Pluronic F-127 and MWCNTs for water desalination. *Desalination*. 2016;397:53-66. doi:10.1016/j.desal.2016.06.019
104. Kim TS, Park HD. Tributyl tetradecyl phosphonium chloride for biofouling control in reverse osmosis processes. *Desalination*. 2015;372:39-46. doi:10.1016/j.desal.2015.06.019
105. Liu Z, Hu Y. Sustainable Antibiofouling Properties of Thin Film Composite Forward Osmosis Membrane with

Rechargeable Silver Nanoparticles Loading. *ACS Appl Mater Interfaces*. 2016;8(33):21666-21673. doi:10.1021/acsami.6b06727

106. Zhang T, Zhu C, Ma H, Li R, Dong B, Liu Y, Li S. Surface modification of APA-TFC membrane with quaternary ammonium cation and salicylaldehyde to improve performance. *J Memb Sci*. 2014;457:88-94. doi:10.1016/j.memsci.2014.01.024
107. Choi W, Chan EP, Park JH, Ahn WG, Jung HW, Hong S, Lee JS, Han JY, Park S, Ko DH, et al. Nanoscale Pillar-Enhanced Tribological Surfaces as Antifouling Membranes. *ACS Appl Mater Interfaces*. 2016;8(45):31433-31441. doi:10.1021/acsami.6b10875
108. Ben-Sasson M, Lu X, Bar-Zeev E, Zodrow KR, Nejati S, Qi G, Giannelis EP, Elimelech M. In situ formation of silver nanoparticles on thin-film composite reverse osmosis membranes for biofouling mitigation. *Water Res*. 2014;62:260-270. doi:10.1016/j.watres.2014.05.049
109. Liu L, Di DYW, Park H, Son M, Hur HG, Choi H. Improved antifouling performance of polyethersulfone (PES) membrane via surface modification by CNTs bound polyelectrolyte multilayers. *RSC Adv*. 2015;5(10):7340-7348. doi:10.1039/c4ra14113j
110. Faria AF, Liu C, Xie M, Perreault F, Nghiem LD, Ma J, Elimelech M. Thin-film composite forward osmosis membranes functionalized with graphene oxide–silver nanocomposites for biofouling control. *J Memb Sci*. 2017;525(October 2016):146-156. doi:10.1016/j.memsci.2016.10.040
111. Liu Z, Qi L, An X, Liu C, Hu Y. Surface Engineering of Thin Film Composite Polyamide Membranes with Silver Nanoparticles through Layer-by-Layer Interfacial Polymerization for Antibacterial Properties. *ACS Appl Mater Interfaces*. 2017;9(46):40987-40997. doi:10.1021/acsami.7b12314
112. Wu J, Yu C, Li Q. Novel regenerable antimicrobial nanocomposite membranes: Effect of silver loading and valence state. *J Memb Sci*. 2017;531(July 2016):68-76. doi:10.1016/j.memsci.2017.02.047
113. Ray JR, Tadepalli S, Nergiz SZ, Liu KK, You L, Tang Y, Singamaneni S, Jun YS. Hydrophilic, bactericidal nanoheater-enabled reverse osmosis membranes to improve fouling resistance. *ACS Appl Mater Interfaces*. 2015;7(21):11117-11126. doi:10.1021/am509174j
114. Wang J, Wang Z, Wang J, Wang S. Improving the water flux and bio-fouling resistance of reverse osmosis (RO) membrane through surface modification by zwitterionic polymer. *J Memb Sci*. 2015;493:188-199. doi:10.1016/j.memsci.2015.06.036
115. Huang L, Zhao S, Wang Z, Wu J, Wang J, Wang S. In situ immobilization of silver nanoparticles for improving permeability, antifouling and anti-bacterial properties of ultrafiltration membrane. *J Memb Sci*. 2016;499:269-281. doi:10.1016/j.memsci.2015.10.055
116. Yang Z, Wu Y, Wang J, Cao B, Tang CY. In situ reduction of silver by polydopamine: A novel antimicrobial modification of a thin-film composite polyamide membrane. *Environ Sci Technol*. 2016;50(17):9543-9550. doi:10.1021/acs.est.6b01867
117. Ben-Sasson M, Lu X, Nejati S, Jaramillo H, Elimelech M. In situ surface functionalization of reverse osmosis membranes with biocidal copper nanoparticles. *Desalination*. 2016;388:1-8. doi:10.1016/j.desal.2016.03.005
118. Mukherjee M, De S. Investigation of antifouling and disinfection potential of chitosan coated iron oxide-PAN hollow fiber membrane using Gram-positive and Gram-negative bacteria. *Mater Sci Eng C*. 2017;75:133-148. doi:10.1016/j.msec.2017.02.039
119. Singh SP, Li Y, Be'Er A, Oren Y, Tour JM, Arnusch CJ. Laser-Induced Graphene Layers and Electrodes Prevents Microbial Fouling and Exerts Antimicrobial Action. *ACS Appl Mater Interfaces*. 2017;9(21):18238-18247. doi:10.1021/acsami.7b04863
120. Kim TS, Park HD. Lauroyl arginate ethyl: An effective antibiofouling agent applicable for reverse osmosis processes producing potable water. *J Memb Sci*. 2016;507:24-33. doi:10.1016/j.memsci.2016.01.056
121. Shao F, Xu C, Ji W, Dong H, Sun Q, Yu L, Dong L. Layer-by-layer self-assembly TiO2 and graphene oxide on polyamide reverse osmosis membranes with improved membrane durability. *Desalination*. 2017;423(September):21-29. doi:10.1016/j.desal.2017.09.007
122. Huang X, Marsh KL, McVerry BT, Hoek EMV, Kaner RB. Low-Fouling Antibacterial Reverse Osmosis Membranes via Surface Grafting of Graphene Oxide. *ACS Appl Mater Interfaces*. 2016;8(23):14334-14338. doi:10.1021/acsami.6b05293
123. Zhang R, Su Y, Zhou L, Zhou T, Zhao X, Li Y, Liu Y, Jiang Z. Manipulating the multifunctionalities of polydopamine to prepare high-flux anti-biofouling composite nanofiltration membranes. *RSC Adv*. 2016;6(39):32863-32873. doi:10.1039/c6ra04458a

124. Zirehpour A, Rahimpour A, Arabi Shamsabadi A, Sharifian MG, Soroush M. Mitigation of Thin-Film Composite Membrane Biofouling via Immobilizing Nano-Sized Biocidal Reservoirs in the Membrane Active Layer. *Environ Sci Technol*. 2017;51(10):5511-5522. doi:10.1021/acs.est.7b00782
125. Choi YS, Kang H, Kim DG, Cha SH, Lee JC. Mussel-inspired dopamine- and plant-based cardanol-containing polymer coatings for multifunctional filtration membranes. *ACS Appl Mater Interfaces*. 2014;6(23):21297-21307. doi:10.1021/am506263s
126. Yu J, Shin GA, Oh BS, Kye J Il, Yoon J. N-chlorosuccinimide as a novel agent for biofouling control in the polyamide reverse osmosis membrane process. *Desalination*. 2015;357:1-7. doi:10.1016/j.desal.2014.11.004
127. Yu Z, Zhao Y, Gao B, Liu X, Jia L, Zhao F, Ma J. Performance of novel a Ag-n-TiO2/PVC reinforced hollow fiber membrane applied in water purification: In situ antibacterial properties and resistance to biofouling. *RSC Adv*. 2015;5(118):97320-97329. doi:10.1039/c5ra18185b
128. Li L, Yan G, Wang H, Chu Z, Li Z, Ling Y, Wu T. Denitrification and microbial community in MBBR using A. donax as carbon source and biofilm carriers for reverse osmosis concentrate treatment. *J Environ Sci (China)*. 2019;84:133-143. doi:10.1016/j.jes.2019.04.030
129. Huang S, Voutchkov N, Jiang S. Balancing carbon, nitrogen and phosphorus concentration in seawater as a strategy to prevent accelerated membrane biofouling. *Water Res*. 2019;165:114978. doi:10.1016/j.watres.2019.114978
130. Jeong D, Lee CH, Lee S, Bae H. Intermittent chlorination shifts the marine biofilm population on reverse osmosis membranes Intermittent chlorination shifts the marine biofilm population on reverse osmosis membranes. 2019;(January 2020). doi:10.12989/mwt.2019.10.6.395
131. Li Y, Li M, Xiao K, Huang X. Reverse osmosis membrane autopsy in coal chemical wastewater treatment: Evidences of spatially heterogeneous fouling and organic-inorganic synergistic effect. *J Clean Prod*. 2020;246:118964. doi:10.1016/j.jclepro.2019.118964
132. Chang H, Liu B, Wang H, Zhang SY, Chen S, Tiraferri A, Tang YQ. Evaluating the performance of gravity-driven membrane filtration as desalination pretreatment of shale gas flowback and produced water. *J Memb Sci*. 2019;587(June):117187. doi:10.1016/j.memsci.2019.117187
133. Lee S, Suwarno SR, Quek BWH, Kim L, Wu B, Chong TH. A comparison of gravity-driven membrane (GDM) reactor and biofiltration + GDM reactor for seawater reverse osmosis desalination pretreatment. *Water Res*. 2019;154:72-83. doi:10.1016/j.watres.2019.01.044
134. Wang YH, Wu YH, Tong X, Yu T, Peng L, Bai Y, Zhao XH, Huo ZY, Ikuno N, Hu HY. Chlorine disinfection significantly aggravated the biofouling of reverse osmosis membrane used for municipal wastewater reclamation. *Water Res*. 2019;154:246-257. doi:10.1016/j.watres.2019.02.008
135. Nagaraj V, Skillman L, Li D, Xie Z, Ho G. Culturable bacteria from a full-scale desalination plant: Identification methods, bacterial diversity and selection of models based on membrane-biofilm community. *Desalination*. 2019;457(December 2018):103-114. doi:10.1016/j.desal.2019.01.028
136. Rehman ZU, Ali M, Iftikhar H, Leiknes ToO. Genome-resolved metagenomic analysis reveals roles of microbial community members in full-scale seawater reverse osmosis plant. *Water Res*. 2019;149:263-271. doi:10.1016/j.watres.2018.11.012
137. Inaba T, Hori T, Aizawa H, Sato Y, Ogata A, Habe H. Microbiomes and chemical components of feed water and membrane-attached biofilm in reverse osmosis system to treat membrane bioreactor effluents. *Sci Rep*. 2018;8(1):1-11. doi:10.1038/s41598-018-35156-2
138. Martínez-Campos S, Redondo-Nieto M, Shang J, Peña N, Leganés F, Rosal R, Fernandez-Piñas F. Characterization of microbial colonization and diversity in reverse osmosis membrane autopsy. *Desalin Water Treat*. 2018;131(November):9-29. doi:10.5004/dwt.2018.22949
139. Park JW, Lee YJ, Meyer AS, Douterelo I, Maeng SK. Bacterial growth through microfiltration membranes and NOM characteristics in an MF-RO integrated membrane system: Lab-scale and full-scale studies. *Water Res*. 2018;144:36-45. doi:10.1016/j.watres.2018.07.027
140. Zheng L, Yu D, Wang G, Yue Z, Zhang C, Wang Y, Zhang J, Wang J, Liang G, Wei Y. Characteristics and formation mechanism of membrane fouling in a full-scale RO wastewater reclamation process: Membrane autopsy and fouling characterization. *J Memb Sci*. 2018;563(June):843-856. doi:10.1016/j.memsci.2018.06.043
141. Belgini DRB, Dias RS, Siqueira VM, Valadares LAB, Albanese JM, Souza RS, Torres APR, Sousa MP, Silva CC, De Paula SO, Oliveira VM. Culturable bacterial diversity from a feed water of a reverse osmosis system, evaluation of biofilm formation and biocontrol using phages. *World J Microbiol Biotechnol*. 2014;30(10):2689-

2700. doi:10.1007/s11274-014-1693-1
142. Farhat NM, Loubineaud E, Prest EIEC, El-Chakhtoura J, Salles C, Bucs SS, Trampé J, Van den Broek WBP, Van Agtmaal JMC, et al. Application of monochloramine for wastewater reuse: Effect on biostability during transport and biofouling in RO membranes. *J Memb Sci*. 2018;551(September 2017):243-253. doi:10.1016/j.memsci.2018.01.060
143. Shang W, Sun F, Chen L. Nanofiltration fouling propensity caused by wastewater effluent organic matters and surface-water dissolved organic matters. *Environ Technol (United Kingdom)*. 2018;39(15):1914-1925. doi:10.1080/09593330.2017.1344324
144. Leddy MB, Hasan NA, Subramanian P, Heberling C, Cotruvo J, Colwell RR. Characterization of Microbial Signatures From Advanced Treated Wastewater Biofilms. *J Am Water Works Assoc*. 2017;109(11):E503-E512. doi:10.5942/jawwa.2017.109.0116
145. Yang Y, Cheng D, Li Y, Yu L, Gin KYH, Chen JP, Reinhard M. Effects of monochloramine and hydrogen peroxide on the bacterial community shifts in biologically treated wastewater. *Chemosphere*. 2017;189:399-406. doi:10.1016/j.chemosphere.2017.09.087
146. Jeong S, Cho K, Jeong D, Lee S, Leiknes TO, Vigneswaran S, Bae H. Effect of engineered environment on microbial community structure in biofilter and biofilm on reverse osmosis membrane. *Water Res*. 2017;124:227-237. doi:10.1016/j.watres.2017.07.064
147. Zhang C, Zhan S, Wang J, Liu Z, You H, Jia Y. Isolation and characterization of seven quorum quenching bacteria for biofouling control in MBR. *Clean Technol Environ Policy*. 2017;19(4):991-1001. doi:10.1007/s10098-016-1294-9
148. Chamberland J, Lessard MH, Doyen A, Labrie S, Pouliot Y. A sequencing approach targeting the 16S rRNA gene unravels the biofilm composition of spiral-wound membranes used in the dairy industry. *Dairy Sci Technol*. 2017;96(6):827-843. doi:10.1007/s13594-016-0305-2
149. Al Ashhab A, Sweity A, Bayramoglu B, Herzberg M, Gillor O. Biofouling of reverse osmosis membranes: effects of cleaning on biofilm microbial communities, membrane performance, and adherence of extracellular polymeric substances. *Biofouling*. 2017;33(5):397-409. doi:10.1080/08927014.2017.1318382
150. Cho K, Jeong S, Kim H, Choi K, Lee S, Bae H. Simultaneous dechlorination and disinfection using vacuum UV irradiation for SWRO process. *Desalination*. 2016;398:22-29. doi:10.1016/j.desal.2016.07.012
151. Sala-Comorera L, Blanch AR, Vilaró C, Galofré B, García-Aljaro C. Pseudomonas-related populations associated with reverse osmosis in drinking water treatment. *J Environ Manage*. 2016;182:335-341. doi:10.1016/j.jenvman.2016.07.089
152. Ishizaki S, Fukushima T, Ishii S, Okabe S. Membrane fouling potentials and cellular properties of bacteria isolated from fouled membranes in a MBR treating municipal wastewater. *Water Res*. 2016;100:448-457. doi:10.1016/j.watres.2016.05.027
153. Li X, Sun S, Badgley BD, He Z. Long-term performance and microbial community characterization of an osmotic anammox system for removing reverse-fluxed ammonium. *Bioresour Technol*. 2016;211:628-635. doi:10.1016/j.biortech.2016.03.137
154. Belila A, El-Chakhtoura J, Otaibi N, Muyzer G, Gonzalez-Gil G, Saikaly PE, van Loosdrecht MCM, Vrouwenvelder JS. Bacterial community structure and variation in a full-scale seawater desalination plant for drinking water production. *Water Res*. 2016;94:62-72. doi:10.1016/j.watres.2016.02.039
155. Levi A, Bar-Zeev E, Elifantz H, Berman T, Berman-Frank I. Characterization of microbial communities in water and biofilms along a large scale SWRO desalination facility: Site-specific prerequisite for biofouling treatments. *Desalination*. 2016;378:44-52. doi:10.1016/j.desal.2015.09.023
156. Khan MT, Hong PY, Nada N, Croue JP. Does chlorination of seawater reverse osmosis membranes control biofouling? *Water Res*. 2015;78:84-97. doi:10.1016/j.watres.2015.03.029
157. Barnes RJ, Low JH, Bandi RR, Tay M, Chua F, Aung T, Fane AG, Kjelleberg S, Rice SA. Nitric oxide treatment for the control of reverse osmosis membrane biofouling. *Appl Environ Microbiol*. 2015;81(7):2515-2524. doi:10.1128/AEM.03404-14
158. Ferrera I, Mas J, Taberna E, Sanz J, Sánchez O. Biological support media influence the bacterial biofouling community in reverse osmosis water reclamation demonstration plants. *Biofouling*. 2015;31(2):173-180. doi:10.1080/08927014.2015.1012640
159. Al Ashhab A, Herzberg M, Gillor O. Biofouling of reverse-osmosis membranes during tertiary wastewater desalination: Microbial community composition. *Water Res*. 2014;50:341-349. doi:10.1016/j.watres.2013.10.044

160. Kim IS, Lee J, Kim SJ, Yu HW, Jang A. Comparative pyrosequencing analysis of bacterial community change in biofilm formed on seawater reverse osmosis membrane. *Environ Technol (United Kingdom)*. 2014;35(2):125-136. doi:10.1080/09593330.2013.817445

161. Khan MT, De O. Manes CL, Aubry C, Gutierrez L, Croue JP. Kinetic study of seawater reverse osmosis membrane fouling. *Environ Sci Technol*. 2013;47(19):10884-10894. doi:10.1021/es402138e

162. Ayache C, Manes C, Pidou M, Croué JP, Gernjak W. Microbial community analysis of fouled reverse osmosis membranes used in water recycling. *Water Res*. 2013;47(10):3291-3299. doi:10.1016/j.watres.2013.03.006

163. Khan MT, Manes CL de O, Aubry C, Croué JP. Source water quality shaping different fouling scenarios in a full-scale desalination plant at the Red Sea. *Water Res*. 2013;47(2):558-568. doi:10.1016/j.watres.2012.10.017

164. Chun Y, Ha PT, Powell L, Lee J, Kim D, Choi D, Lovitt, RW, Kim IS, Mitra SS, Chang IS. Exploring microbial communities and differences of cartridge filters (CFs) and reverse osmosis (RO) membranes for seawater desalination processes. *Desalination*. 2012;298(January 2018):85-92. doi:10.1016/j.desal.2012.05.007

165. Chiellini C, Iannelli R, Modeo L, Bianchi V, Petroni G. Biofouling of reverse osmosis membranes used in river water purification for drinking purposes: analysis of microbial populations. *Biofouling*. 2012;28(9):969-984. doi:10.1080/08927014.2012.724679

166. Zhang M, Jiang S, Tanuwidjaja D, Voutchkov N, Hoek EMV, Cai B. Composition and variability of biofouling organisms in seawater reverse osmosis desalination plants. *Appl Environ Microbiol*. 2011;77(13):4390-4398. doi:10.1128/AEM.00122-11

167. Bae H, Kim H, Jeong S, Lee S. Changes in the relative abundance of biofilm-forming bacteria by conventional sand-filtration and microfiltration as pretreatments for seawater reverse osmosis desalination. *Desalination*. 2011;273(2-3):258-266. doi:10.1016/j.desal.2010.12.030

168. Lee J, Kim IS. Microbial community in seawater reverse osmosis and rapid diagnosis of membrane biofouling. *Desalination*. 2011;273(1):118-126. doi:10.1016/j.desal.2010.12.005

169. de CL, West N, Rapenne S, Lebaron P. Dynamic bacterial communities on reverse-osmosis membranes in a full-scale desalination plant. *Biofouling*. 2011;27(1):47-58. doi:10.1080/08927014.2010.536980

170. Lee J, Jung JY, Kim S, Chang IS, Mitra SS, Kim IS. Selection of the most problematic biofoulant in fouled RO membrane and the seawater intake to develop biosensors for membrane biofouling. *Desalination*. 2009;247(1-3):125-136. doi:10.1016/j.desal.2008.12.018

171. Ivnitsky H, Katz I, Minz D, Volvovic G, Shimoni E, Kesselman E, Semiat R, Dosoretz CG. Bacterial community composition and structure of biofilms developing on nanofiltration membranes applied to wastewater treatment. *Water Res*. 2007;41(17):3924-3935. doi:10.1016/j.watres.2007.05.021

172. Chen CL, Liu WT, Chong ML, Wong MT, Ong SL, Seah H, Ng WJ. Community structure of microbial biofilms associated with membrane-based water purification processes as revealed using a polyphasic approach. *Appl Microbiol Biotechnol*. 2004;63(4):466-473. doi:10.1007/s00253-003-1286-7

173. Chee MP, Liu WT. Community structure analysis of reverse osmosis membrane biofilms and the significance of Rhizobiales bacteria in biofouling. *Environ Sci Technol*. 2007;41(13):4728-4734. doi:10.1021/es0701614
